# Supplementary material for: A bioavailable strontium (87Sr/86Sr) isoscape for Aotearoa New Zealand: Implications for food forensics and biosecurity
Source: PLoS One. 2022 Mar 16;17(3):e0264458. doi: 10.1371/journal.pone.0264458 (PMC8926269; doi:10.1371/journal.pone.0264458)
Supplement: S1 File — S1.1. Aotearoa Plant Sample Preparation. S1.2. Aotearoa Topsoil Sample Preparation. S1.3. Collected Plant and Topsoil MC-ICP-MS Analysis. S1.4. Additional Plant and Topsoil Preparation and Analysis. S1.5. Cow Milk Sample Preparation and Analysis. (DOCX) [file pone.0264458.s001.docx]

# S1. SAMPLE PREPARATION AND ANALYSIS

# S1.1. Aotearoa Plant Sample Preparation

Plant samples collected from Aotearoa by RTK, WDKS, and RLK were prepared by RTK under the supervision of lab technician, David Barr, in the class-10, ultra-clean laboratory in the Department of Geology, University of Otago following the Centre for Trace Element Analysis standard preparation and analytical methods. All plant samples were collected on public roads which provides access to any member of the public. Permits are not necessary to collect samples of plants (leaves and stems) on public roadways in Aotearoa New Zealand. Plant samples were cut into 1-2 cm sized segments and approximately 1 g of dried plant material was weighed into acid-washed, labelled DigiTUBEs (SCP Science, Canada). Then, plants were leached by adding 10-20 mL of 2% HNO_3_ solution to each DigiTUBE and soaked for 10-15 minutes. Once leached, the supernatant acid was removed from each DigiTUBE using an acid-washed 5 mL pipette and then the plant sample was rinsed with milli-Q (MQ) water three times (MQ was removed with pipette as well). Uncapped DigiTUBEs were then placed on the 54-well HotBlock system (Environmental Express, Charleston, South Carolina, USA) to dry at 90º C for approximately 30 minutes or until dry. Once dried, 500 mg of each sample were measured into clean MARS 6 microwave PTFE (Polytetrafluoroethylene) pressure vessels (CEM Corporation, North Carolina, USA). Then, 10 mL of quartz distilled (QD) HNO_3_ and 1 mL of 30% H_2_O_2_ were added to each tube, capped tightly using a torque block, and microwave digested using the MARS 6 (CEM Corporation, North Carolina, USA) 6 pre-programmed “Plant Digestion” setting (800 W of power, 17-minute preheating time, 43 minutes of consistent heat with a maximum temperature of 200 °C). Once digested, the resulting solution was transferred into acid-washed, labelled DigiTUBEs. MQ was added to the microwave tube using a squeeze bottle and swirled to collect any residual digested solution and then added to the labelled DigiTUBE with the rest of the digested sample. Enough MQ was added to bring the final solution up to 50 mL. Then, 20 μL were subsampled from each of the digested solutions, transferred into acid-washed, polyethylene ICP-MS auto-sampler vials, and analyzed by ICP-AES to check the strontium and other trace element concentrations before moving to the next step. Ten duplicate plant samples were digested and added to total samples.

After ICP-AES analysis, the lab technician calculated how much of each plant solution was required to dry down to obtain a strontium concentration of ~65 mg/kg (parts per million, ppm). The required volumes for each sample were pipetted into clean Perfluoroalkoxy alkanes or PFA vials (Savillex, Eden Prairie, Minnesota, USA), transferred into the clean lab, and set on hotplates inside vented evaporation stations to dry down. Once dry, 5 mL of 2N HNO_3_ solution was added to each and set on hotplate to reflux for 1-2 hours. If the resulting solution was green in color (most were) this indicated that organic material was still present and further digestion was necessary. Therefore, the solution was evaporated at 120º C and 4 mL of QD HNO_3_ was added to each, then capped, and set to reflux for three days at 110º C. After three days, caps were removed, and the solution evaporated. Then, 1 mL QD HNO_3_ and 1 mL of 30% H_2_O_2_ added to each vial, capped, and set to reflux for another two days. After refluxing, solutions were evaporated once more and then brought up to a 2N HNO_3_ solution. If the green color persisted, the same process was repeated until the solution was a clear to light pale green color, indicating that enough of the organic material had been digested to move to the Sr separation step. Once prepped, solutions were transferred into acid-washed, labelled ICP-MS vials and transferred to the prepFAST-MC instrument (Elemental Scientific; ESI, Omaha, Nebraska, USA) for the automated strontium separation chemistry as described by Wijenayake [9]. The automated ion-exchange chromatography method uses a 3-mL column (ESI, Part number CF-MC-SrCa-3000) filled with DGA (diglycolamide) resin (TrisKem International, Bruz, France).

**S1.2. Aotearoa Topsoil Sample Preparation**

Through collaboration with the Crown research institute GNS Science, topsoil samples (0-20 cm) from Aotearoa were added to this project. In total, 72 samples from the Otago, Southland, and Nelson regions were collected from the GNS storage facility in Avalon and analyzed for strontium isotope ratios. Soil samples were obtained after written or verbal permission from the private, commercial or government landowner with permissions held by GNS Science and/or New Zealand Petroleum and Minerals. Topsoil samples were collected by hand auger and the sub-2-mm portion was retained after sieving and drying at 40 °C [55,97]. Subsequently, samples were sifted and stored in labeled plastic vials until used for this study. Once collected from storage, samples were transported to the University of Otago and a 4 g subsample was extracted from each vial and placed into labelled, polyethylene ICP-MS auto-sampler vials.

Following methodologies outlined in Willmes et al. [86] and the International Organization for Standardization protocol (ISO Standard No. 19730:2008), a 1 g aliquot was subsampled into 15 mL centrifuge tubes and leached in 2.5 mL of 1 M ammonium nitrate (NH_4_NO_3_) solution and agitated overnight. We could not access an “over-under” shaker as suggested in the ISO and instead used a side-to-side, upright shaker. Once shaken, samples were centrifuged at 3000 rpm for 15 minutes using an Eppendorf Centrifuge 5810. Then, using an acid-washed pipette, 1 mL of supernatant was extracted from each sample, pipetted into PFA vials (Savillex, Eden Prairie, Minnesota, USA), and set to evaporate (uncapped) for 2-4 hours at 110°C. Once dry, 50 μL of QD HNO_3_ acid and 2 mL of MQ water was added to each vial and set back on hotplate to dissolve for 2 hours at 110°C. Once dissolved, 20 μL were pipetted from each solution, transferred into acid-washed ICP-MS vials, and analyzed using ICP-AES to check the strontium and other trace element concentrations.

The strontium contents of the soil solutions were low compared to the plant samples, so once samples were brought up to a 3M HNO_3_ solution, the strontium was manually separated as described by Pin and Bassin [104] using an Eichrom Sr specific resin instead of using the automated prepFAST-MC (Elemental Scientific; ESI, Omaha, Nebraska, USA). Once isolated from other potential interfering elements like rubidium, the sample solutions were evaporated to dryness on a hotplate for 4-6 hours at 110°C. Once evaporated, 50 μL of QD HNO_3_ acid and 2 mL of MQ water was added to each vial and set back on hotplate to dissolve for two hours at 110°C. The resulting 2% HNO_3_ solution were subsampled once more to check strontium concentration by ICP-AES and then diluted accordingly (aiming for 200 ppb Sr concentration) for analysis by Nu Plasma HR MC-ICP-MS.

**S1.3. Collected Plant and Topsoil MC-ICP-MS Analysis**

Once all plant and topsoil samples had gone through the strontium separation step, all were dried down and brought up to 2 mL volume through addition of 2% HNO_3_ solution. Strontium concentrations were checked using ICP-AES and each sample was diluted to 200 ppb before being analyzed by the Nu Plasma HR MC-ICP-MS. Data were normalized using repeated measurement of two in-house lab references, NIST SRM 987 and HPS, that bracketed every six samples to monitor the accuracy and reproducibility of the measurements. The in-house, long-term reference value for NIST SRM 987 is 0.71025 ± 0.00002 (2 SD, n > 200) and for HPS it is 0.70762 ± 0.00003 (2 SD, n=189). After analyzing all plant and topsoil samples, we obtained averages of 0.71025 ± 0.00002 (2 SD, n=70) for NIST SRM 987 and 0.70761 ± 0.00015 (2 SD, n=58) for HPS. Any instrumental mass fractionation present was corrected using repeated measurement of ^86^Sr/^88^Sr, expected to be 0.1194. Procedural blanks were run with each batch of 6 samples and all yielded negligible Sr levels of < 250 pg.

**S1.4. Additional Plant and Topsoil Preparation and Analysis**

The “additional plant” data were generated by the Bio-Protection Research Centre’s project, B3 D19.8, in collaboration with Better Border Biosecurity (www.b3nz.org.nz/). Where on private land, the plant and soil samples from Holder 2012, PBCRC2111 unpub, and B3_PH-KA were collected with the permission of the landholders. A few leaf samples in B3_PH-KA were collected from publicly accessible amenity planting, for which collection permits are not required. Ten mature leaves were taken from around the circumference of each tree to avoid directional bias. The leaves were not cleaned, but only those free from visible dust contamination and pest infestation were selected. Sterile gloves were used when collecting, to avoid contaminating the samples. The samples were sealed in breathable, single-use, autoclave envelopes, and field preservation was achieved by desiccation over silica gel. The samples were then dried at 40º C for a minimum of 3 weeks, in the original autoclave envelope; and stored in the oven until being powdered in a cleaned agate mortar. Approximately 0.5 g of powdered plant material was weighed into acid-washed MARS 6 microwave reaction tubes, and 10 mL of quartz distilled (QD) HNO_3_ was added to each tube. The samples were microwave digested using the MARS 6 pre-programmed “Plant Digestion” setting at 200º C. Once digested, the resulting solution was transferred into acid-washed, labelled DigiTUBEs. MilliQ water (MQ, 18.1 Ω) was then added to bring the final solution up to 50 mL. A 20 μL aliquot was taken from each of the digested solutions, and transferred into acid-washed, polyethylene ICP-MS auto-sampler vials for Trace Element analysis at the Centre for Trace Element Analysis, Otago University, Dunedin. The remainder of the samples were then dried down. Sr isotope measurement of these samples was conducted using the MS-MS ICP-MS method of Murphy et al. [69], at Queensland University of Technology, Brisbane.

The “additional soil” data were generated by Holder [105] and the unpublished PBCRC2111 project Armstrong et al. [106]. The topsoil collection in both studies followed the protocol established in the [S1.6.1] project. Namely, a single top-soil sample was collected at each site, from the root-ball area, below the leaf litter zone. A stainless-steel trowel was used to collect 50 mL of soil into glass tubes. All visible root and plant debris were removed from the soils using fine forceps. The soil was then dried at 80°C in labelled glass vials for at least 48 hours and then gently crushed using a clean mortar and pestle until even, medium sized aggregates were achieved. Approximately 25 grams of the fine material was stored in numbered sealed glass vials for subsequent digestion and archiving. Holder [105] used a total recoverable soil digest. 5 mL 15 M Seastar HNO_3_ and 1 mL Aristar (30%) H_2_O_2_ were added to weighed (≈ 0.5 g) soil in 55 mL microwave digestion vessels. The lids were screwed down loosely, and the samples allowed to pre-digest for 24 – 48 h. The vessels were then subject to a 10 min ramp to 175º C, then held at 175° C for 20 min, and then allowed to cool for 2 h. The solutions were transferred to cleaned 50 mL c-tubes and diluted to ≈ 20 mL with MQ water. The tubes were then spun at 3300 rpm for 10 min. An aliquot was taken off each of the samples for trace element analysis. The remainder of the solution was centrifuged again at 3300 rpm for 10 min, then approximately 9 mL was gently pipetted to clean 23 mL Savillex PFA vials and dried down for storage prior to column chemistry.

After freeze drying and pulverising about 5g of soil samples were weighed in 50 mL centrifuge tubes and 1M ammonium acetate solution was added to total V about 50 mL. The samples were shaken overnight, centrifuged at 35 RPM for 15 min and 10 mL aliquots were taken from the middle of solutions and placed in new 10 mL centrifuge tubes. The samples (in 10 mL tubes) were centrifuged again at the same condition, 1 mL aliquots were taken and removed to a new set of 10 mL tubes and topped with 1% double dist. HNO_3_ to 10 mL. Solution weights throughout were used to calculate dilution factors. The extracted solution was then digested using the Ultrawave microwave method, following the recommended recipe for soils, namely UW-25 and 26. An aliquot was taken off each of the samples for trace element analysis. The remaining portion of the solutions was transferred to clean 23 mL Savillex beakers and dried down for storage prior to column chemistry.

Sr separation column procedures used by both projects were those as described by Pin & Bassin [104]. Sr isotope measurement of the samples from Holder [105] was conducted using a Nu Plasma-HR MC ICP-MS (Nu Instruments Ltd., UK) at the Centre for Trace Element Analysis, Otago University, Dunedin (standard = NIST SRM 987, n = 19, ⁸⁷Sr/⁸⁶Sr 0.710274 ±0.000023 (2 SD)); and the Armstrong et al. [106] samples measured using Isotopx Phoenix TIMS (thermal ionization mass spectrometry) at the University of Adelaide (NIST SRM 987, n = 7, ⁸⁷Sr/⁸⁶Sr = 0.710245 ± 0.000008 (2 SD)).

**S1.5. Cow Milk Sample Preparation and Analysis**

Cow milk samples used for this study were obtained from cattle that had been prescribed a controlled feeding regime where they were pasture-fed on-site and not provided with any additional dietary supplements. Cow milk samples were received freeze-dried after being centrifuged at approximately 1000 rpm for 10 minutes, then skimmed. Each sample was accompanied by metadata including the latitude, longitude, and altitude of the sampling location, sampling period, agricultural practice, and feed type were provided. Freeze-dried cow milk powders were crushed to a fine powder using plastic stirrer tips, then mixed thoroughly to homogenise. Acid washed plastic containers and utensils were used during the sample collection and processing to avoid contamination.

All chemical preparation was performed in the class 10, ultra-clean laboratory suite of the Centre for Trace Element Analysis of the University of Otago. For each milk powder digest, 0.5 g was weighed and transferred into PTFE (Polytetrafluoroethylene) pressure vessels (CEM Corporation, North Carolina, USA). Then, 1 mL of MQ H_2_O was added, followed by the addition of 5 mL of 14 M HNO_3_ and 1 mL of H_2_O_2._ The vessels were sealed tightly using a torque block and left to sit for approximately 1 hour before being microwave-digested in the Microwave-Assisted Reaction System (MARS 6, CEM Corporation, North Carolina, USA). The microwave was operated with a power setting of 800 W with a maximum temperature of 200 °C attained over a ramping time of 17 min, followed by a holding time of 43 minutes. Once completed, digested milk powder solutions were transferred into 50 mL DigiTUBEs (SCP Science, Canada) and diluted to 50 mL with MQ H_2_O. Then, a 100 uL aliquot was transferred to a 5 mL polyethylene ICP-MS auto-sampler vial, then diluted to 5 ml with 2% v/v HNO_3_ in preparation for elemental concentration analysis by quadrupole ICP-MS (Q-ICP-MS) to quantify the content of Sr and other elements that could cause interferences during Sr isotope measurement.

Extraction and purification of Sr for each sample was completed using a prepFAST-MC instrument (Elemental Scientific; ESI, Omaha, Nebraska, USA). Each digested sample was evaporated to dryness using the 54-well HotBlock system (Environmental Express, Charleston, South Carolina, USA) by heating to 80 °C for about 8 hours. Once evaporated, samples were redissolved in 10 mL of 14 M HNO3 (Q-HNO3) and refluxed for 1 - 1.5 hours at 92 °C using the HotBlock to remove residual organic matter. Samples were evaporated once more on the HotBlock and were then redissolved in 7.5 mL of 2 M HNO3, of which 5 mL was removed and transferred to an ICP-MS auto-sampler vial before being placed in the prepFAST-MC instrument.

Automated extraction and purification of Sr was performed by the prepFAST-MC in batches of 20, each comprising 15 cow milk samples, two blanks, and a single HPS quality control standard. A 3-mL column (ESI, Part number CF-MC-SrCa-3000) filled with DGA (diglycolamide) resin (TrisKem International, Bruz, France) was used for ion-exchange chromatography. A 2 M HNO_3_ solution was used to load each sample onto the resin. Sr was eluted in 5 M HNO_3_ for each sample, removed from the prepFAST-MC, and then evaporated to dryness using the HotBlock. The samples were then redissolved in 2 mL of 2% v/v HNO_3_ in preparation for Sr isotope measurement. Before analysis, a 100 ul aliquot was removed from each sample, transferred into acid-washed ICP-MS auto-sampler vials, and diluted with 2 mL of 2% v/v HNO_3_ for elemental concentration analysis by ICP-MS. This allowed for the determination of Sr recovery, appropriate dilution of samples, and confirmed whether sufficient Rb and Ca had been removed from the samples during ion-exchange separation.

After conducting the matrix separation process using the automated prepFAST-MC, the Sr isotope composition of the eluted Sr fraction of each milk powder digest was measured at the Centre for Trace Element Analysis, University of Otago using a Nu Plasma-HR MC-ICP-MS instrument. The final corrected ^87^Sr/^86^Sr results for all samples and standards were normalized to the composition of NIST SRM 987 with ^87^Sr/^86^Sr_SRM-987_ = 0.710248 [S1.6.2]. This gives rise to an average ^87^Sr/^86^Sr value of 0.710248 ± 0.000016 (2 SD, n = 35) for NIST SRM 987 measurements. An average ^87^Sr/^86^Sr value of 0.707624 ± 0.000026 (2 SD, n = 28) was obtained for the HPS standard, which is in very good agreement with the long-term in-house accepted value of 0.707618 ± 0.000026 (2 SD, n > 300).

**S1.6. REFERENCES**

S1.6.1. TRACE. Standard Operating Procedures: Soil and water sampling. 2005; 1-20.

S1.6.2. McArthur JM. Recent trends in strontium isotope stratigraphy. Terra Nova. 1994;6: 331-58. doi: 10.1111/j.1365-3121.1994.tb00507.x.
