# Supplementary material for: A bioavailable strontium (87Sr/86Sr) isoscape for Aotearoa New Zealand: Implications for food forensics and biosecurity
Source: PLoS One. 2022 Mar 16;17(3):e0264458. doi: 10.1371/journal.pone.0264458 (PMC8926269; doi:10.1371/journal.pone.0264458)
Supplement: S2 File — S2 Table 1. Geological and climatic variables used in random forest regression. D = discrete variable; C = continuous variable. S2 Table 2. “Toprock” Category (#1–67) Descriptions. S2 Fig 1. The top performing covariate “Toprock” selected by VSURF package. The 67 toprock principal surface lithology types have been simplified but all types are detailed in S2 Table 2. Data sourced from LRIS (https://lris.scinfo.org.nz/layer/48065-nzlri-rock/; 2010) [S5.18] and GNS (fault lines shapefile) [S5.20]. The “toprock” shapefile does not include data for Rakiura (Stewart Island) or Wharekauri (Chatham Islands). (DOCX) [file pone.0264458.s002.docx]

# S.2. RANDOM FOREST MODEL AND AUXILIARY VARIABLES

This study follows the methodology published by Bataille et al. [57,93] to create the first ^87^Sr/^86^Sr isoscape for Aotearoa NZ using a random forest model framework. Bataille and colleagues [93] concluded that random forest regression was the best model to use for constructing a ⁸⁷Sr/⁸⁶Sr model due to the increased accuracy and the model’s “flexible framework” that can be adjusted to include any number of environmental variables for an array of spatial extents and resolutions.

Random forests consist of a large ensemble of decorrelated decision trees trained via bootstrap sampling [111,S2.1.1-3]. Each decision tree within the forest has a unique combination of the randomly selected weak predictor variables and a “bagged version” of the training dataset [S2.1.3]. At each node in a tree, the algorithm randomly selects a subset of predictor variables and then chooses the best performing variable to split the node into two, creating a branch [S2.1.2]. This is repeated at each subsequent node in the tree ensuring that all variables are considered when predicting the mean value of the response variable, ^87^Sr/^86^Sr [94,S2.1.1]. The final prediction is estimated by averaging across the decorrelated random forest which reduces the variance and the tendency to overfit the data [S2.1.1-2]. Averaging the prediction outputs of all decision trees reduces makes random forests highly effective when working on large datasets with missing data [S2.1.2].

The random forest R-script provided by Bataille et al. [57,93] optimizes the regression model with the root mean square error (RMSE) and uses five 10-fold repeated cross-validations on 80% of the training data. Additionally, the R-script includes using the Variable Selection Under Random Forest (*VSURF*) package [107] to identify irrelevant and highly predictive variables [57,93,94]. The relationships between the variables selected by the *VSURF* function and the bioavailable ⁸⁷Sr/⁸⁶Sr variability are assessed using variable importance purity measure and partial dependence plots [94]. We use the random forest to predict the bioavailable ^87^Sr/^86^Sr for Aotearoa NZ using the plant, soil, and mammal bioavailable ^87^Sr/^86^Sr dataset and a variety of independent predictor auxiliary variables (S2 Table 1) including a global bedrock ⁸⁷Sr/⁸⁶Sr model [92]. The auxiliary variables are obtained from various sources summarized in Table 1 and represent geological, climatic, and environmental variables that may influence bioavailable ^87^Sr/^86^Sr variability. Most variables were available as global rasters that were trimmed to an Aotearoa NZ extent and projected to New Zealand Transverse Mercator 2000.

**S2 Table 1. Geological and climatic variables used in random forest regression.** D = discrete variable; C = continuous variable

| **Variable** | **Description** | **Resolution** | **Type** | **Source** |
| --- | --- | --- | --- | --- |
| r.m1 | Median bedrock model output | 1 km | D | [93] |
| r.age | Terrane age attribute | 1 km | D | [S2.1.4] |
| r.salt | CCSM.3 (Community Climate System Model) simulation (log transformed) | 1.4° x 1.4° | C | [S2.1.5] |
| r.dust | Multi-models average (log transformed) | 1° x 1° | C |  |
| r.elevation | Shuttle Radar Topography Mission -SRTM (m) | 90 m | C | [S2.1.6] |
| r.cec | Cation exchange capacity | 250 m | C | [S2.1.7] |
| r.ph | Soil pH in H_2_O solution | 250 m | C |  |
| r.clay | Clay (weight %) | 250 m | C |  |
| r.silt | Silt (weight %) | 250 m | C |  |
| r.sand | Sand (weight %) | 250 m | C |  |
| r.orc | Soil organic carbon (weight %) | 250 m | C |  |
| r.bulk | Bulk density (kg m^-3^) | 250 m | C |  |
| r.nitrogen | Nitrogen (cg/kg) | 250 m | C |  |
| r.bouguer | WGM2012_Bouguer model | 2 min | C | [S2.1.9] |
| r.thick | Global soil thickness | 1 km | C | [S2.1.9] |
| r.upland | Global upland hill slope soil thickness | 1 km | C |  |
| r.map | Mean annual precipitation (mm.yr^-1^)  (log transformed) | 30-arc sec | C | [S2.1.10] |
| r.mat | Mean annual temperature (°C) | 30-arc sec | C |  |
| r.GUM | Global unconsolidated sediment map | 1 km | C | [S2.1.11] |
| r.pet | Global potential evapo-transpiration | 30-arc sec | C | [S2.1.12] |
| r.ai | Global Aridity Index | 30-arc sec | C |  |
| r.lc | Global land cover 2009 | 300 m | D | [S2.1.13] |
| n.fert | Global nitrogen fertilization | 30-arc sec | C | [S2.1.14] |
| r.ssa | Multi-models average sea salt wet + dry deposition (kg.ha ^-1^.yr ^-1^) | 1° x 1° | C | [S2.1.15] |
| r.ssaw | Multi-models average sea salt wet deposition (kg.ha ^-1^.yr ^-1^) | 1° x 1° | C |  |
| r.gravel | Topsoil gravel content | 25 m | D | [S2.1.16] |
| r.perm | Permeability profile (m) | 25 m | C |  |
| r.PRD | Potential rooting depth (m) | 25 m | C |  |
| r.toprock | First named entire rock type | 25 m | D |  |
| r.baserock | Principal basement rock classification | 25 m | D |  |
| r.AWD | Annual Water Deficit (mm) | 100 m | C | [S2.1.17] |
| r.gns_age | GNS geological age attribute | 1 km | D | [S2.1.18] |
| r.geounits | GNS geological unit attribute | 2.5 km | D |  |
| r.rockgrp | GNS geological rock group attribute | 2.5 km | D |  |
| r.GNSagemin | GNS geological rock age minimum | 1 km | C |  |
| r.GNSagemax | GNS geological rock age maximum | 1 km | C |  |
| r.coastdist | Distance to closest coastline (km) | 1° x 1° | C | [S2.1.19] |

**S2 Table 2. “Toprock” Category (#1-67) Descriptions**

| # | ID | Description | Island | Rock Type |
| --- | --- | --- | --- | --- |
| 1 | Al | Undifferentiated floodplain alluvium | Both Islands | Sedimentary |
| 2 | Mo | Ashes older than Taupō pumice | North Island | Igneous |
| 3 | Lo | Loess | Both Islands | Sedimentary |
| 4 | Sm | Sandstone or coarse siltstone — massive | Both Islands | Sedimentary |
| 5 | Gw | Greywacke | Both Islands | Sedimentary |
| 6 | Kt | Kaharoa & Taupo ashes | North Island | Igneous |
| 7 | Mj | Mudstone or fine siltstone — jointed | North Island | Sedimentary |
| 8 | Us | Unconsolidated to moderately consolidated clays, silts, sands, tephra & breccias | North Island | Igneous |
| 9 | Vo | Lavas, ignimbrite & other ‘hard’ volcanic rocks | North Island | Igneous |
| 10 | Ac | Argillite — crushed | North Island | Igneous |
| 11 | Ar | Argillite | North Island | Igneous |
| 12 | Mb | Mudstone or fine siltstone — banded | North Island | Sedimentary |
| 13 | Tp | Taupo & Kaharoa breccia & volcanic alluvium | North Island | Igneous |
| 14 | Gr | Gravels | North Island | Sedimentary |
| 15 | Pt | Peat | Both Islands | Sedimentary |
| 16 | Mm | Mudstone or fine siltstone — massive | North Island | Sedimentary |
| 17 | Wb | Sands — windblown | North Island | Sedimentary |
| 18 | St2 | Schist | South Island | Metamorphic |
| 19 | Gw' | Greywacke - deep weathering | Both Islands | Sedimentary |
| 20 | Sb | Sandstone or coarse siltstone — banded | North Island | Sedimentary |
| 21 | Li | Limestone | North Island | Sedimentary |
| 22 | Ms | Mudstone - weakly indurated | South Island | Sedimentary |
| 23 | lak | Lake | Both Islands | Water |
| 24 | Vo' | Lavas, ignimbrite & ‘hard’ volcanic rocks - deep weathering | Both Islands | Igneous |
| 25 | Ss | Sandstone - weakly indurated | South Island | Sedimentary |
| 26 | riv | River | Both Islands | Water |
| 27 | est | Estuary | Both Islands | Water |
| 28 | St1 | Semi-schist | South Island | Metamorphic |
| 29 | Mx | Sheared mixed lithologies | North Island | Sedimentary |
| 30 | Sb' | Sandstone or coarse siltstone — banded - deep weathering | South Island | Sedimentary |
| 31 | Gn | Crystalline intrusive rocks | North Island | Igneous |
| 32 | Cw | Conglomerate - weakly indurated | South Island | Sedimentary |
| 33 | Gs | Gneiss | South Island | Metamorphic |
| 34 | tow | Town, urban area, airport, oxidation pond | Both Islands | Other |
| 35 | Hs | Sandstone - strongly indurated | South Island | Sedimentary |
| 36 | Sm' | Sandstone or coarse siltstone — massive - deep weathering | Both Islands | Sedimentary |
| 37 | ice | Icefield or Glacial Till | Both Islands | Other |
| 38 | Ls | Limestone - strongly indurated | South Island | Sedimentary |
| 39 | Cg | Conglomerate & Breccia | Both Islands | Sedimentary |
| 40 | Ar' | Argillite - strongly indurated - deep weathering | Both Islands | Sedimentary |
| 41 | In | Ancient volcanoes, minor intrusives (dikes & sills) | South Island | Igneous |
| 42 | Vu' | ‘Soft’ volcanic rocks - deep weathering | North Island | Igneous |
| 43 | Ta | Tarawera ash and lapilli | North Island | Igneous |
| 44 | Me | Mudstone — bentonitic | North Island | Sedimentary |
| 45 | Ng | Ngauruhoe ash | North Island | Igneous |
| 46 | La | Lahar deposits | North Island | Igneous |
| 47 | Lp | Lapilli | North Island | Igneous |
| 48 | Us' | Unconsolidated to moderately consolidated clays, silts, sands, tephra & breccias - deep weathering | North Island | Sedimentary |
| 49 | Um | Ultramafics | Both Islands | Igneous |
| 50 | Rm | Rotomahana mud | North Island | Igneous |
| 51 | Sc | Scoria | North Island | Igneous |
| 52 | Ma | Marble | South Island | Metamorphic |
| 53 | Mm' | Mudstone or fine siltstone — massive - deep weathering | North Island | Sedimentary |
| 54 | qua | Quarry, mine, other earthworks | Both Islands | Other |
| 55 | Cg' | Conglomerate - strongly indurated - deep weathering | Both Islands | Sedimentary |
| 56 | Fy | Interbedded sandstone & mudstone | South Island | Sedimentary |
| 57 | Ac' | Argillite — crushed - deep weathering | North Island | Sedimentary |
| 58 | Mb' | Mudstone or fine siltstone — banded - deep weathering | North Island | Sedimentary |
| 59 | Mj' | Mudstone or fine siltstone — jointed - deep weathering | North Island | Sedimentary |
| 60 | Af | Fine Alluvium | Both Islands | Sedimentary |
| 61 | Ft | Breccias older than Taupo breccia | North Island | Sedimentary |
| 62 | Tb | Pyroclastics (ash & lapilli) | South Island | Igneous |
| 63 | Gn' | Crystalline intrusive rocks - deep weathering | Both Islands | Igneous |
| 64 | Sc' | Scoria - deep weathering | North Island | Igneous |
| 65 | Vu | ‘Soft’ volcanic rocks | North Island | Igneous |
| 66 | riv | River | Both Islands | Water |
| 67 | Mx' | Sheared mixed lithologies - deep weathering | North Island | Sedimentary |

Source: Land Resource Information System Spatial Data Layers Data Dictionary [S2.1.16].


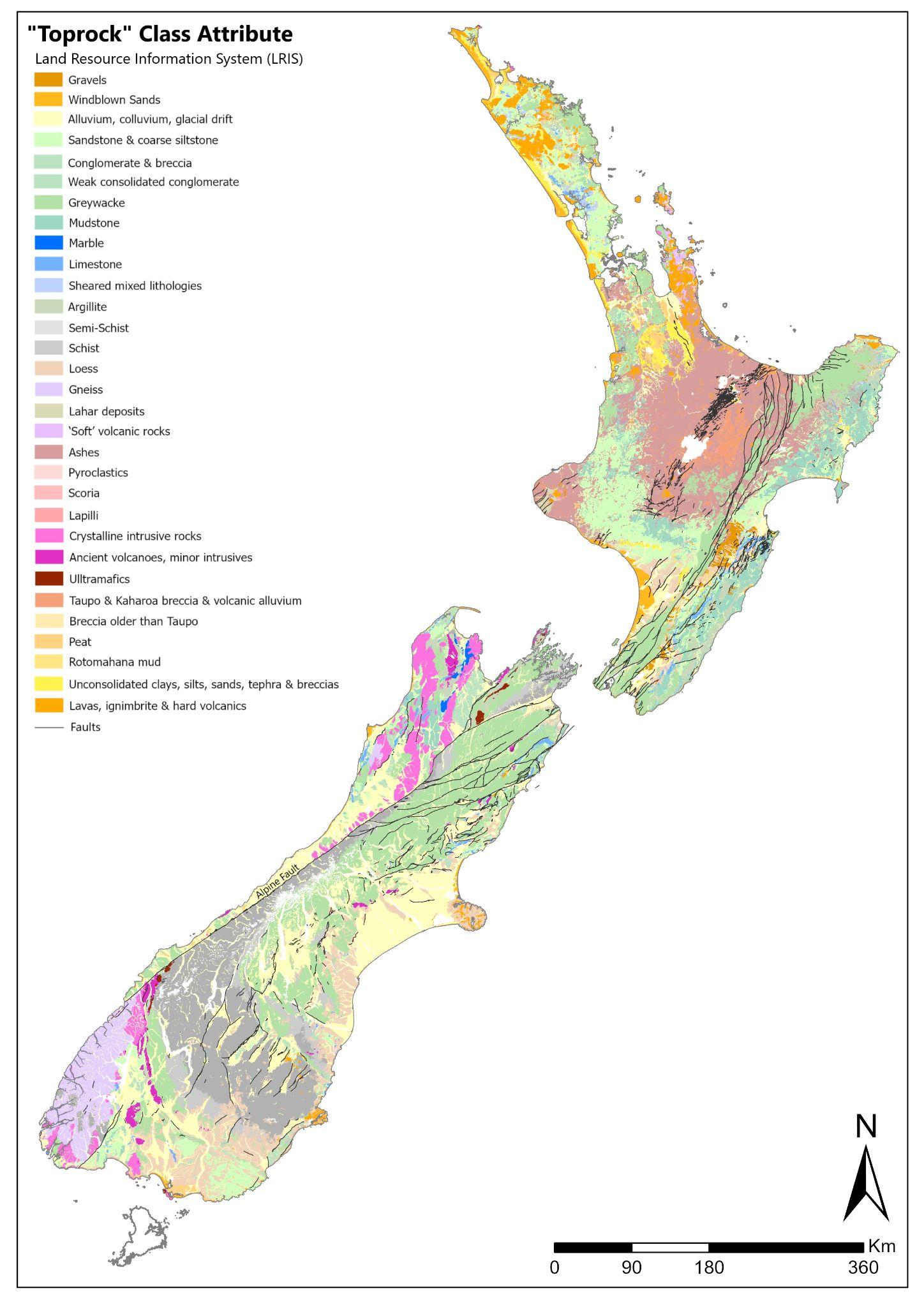


**S2 Fig 1.** **The top performing covariate “Toprock” selected by *VSURF* package.** The 67 toprock principal surface lithology types have been simplified but all types are detailed in S2 Table 2. Data sourced from LRIS (<https://lris.scinfo.org.nz/layer/48065-nzlri-rock/>; 2010) [S2.1.16] and GNS (fault lines shapefile) [S2.1.18]. The “toprock” shapefile does not include data for Rakiura (Stewart Island) or Wharekauri (Chatham Islands).

**S2.1. REFERENCES**

S2.1.1. Ayyadevara VK. Pro Machine Learning Algorithms: A Hands-On Approach to Implementing Algorithms in Python and R. Berkeley: Apress; 2018. doi: 10.1007/978-1-4842-3564-5.

S2.1.2. Awad M, Khanna R. Efficient Learning Machines: theories, concepts, and applications for engineers and system designers. Springer Nature: New York; 2015.

S2.1.3. Vaysse K, Lagacherie P. Using quantile regression forest to estimate uncertainty of digital soil mapping products. Geoderma. 2017;291: 55-64. doi: 10.1016/j.geoderma.2016.12.017.

S2.1.4. Mooney WD, Laske G, Masters TG. CRUST 5.1: A global crustal model at 5Ê × 5Ê. J Geophys Res Solid Earth. 1998;103: 727-747. doi: 10.1029/97JB02122.

S2.1.5. Mahowald NM, Muhs DR, Levis S, Rasch PJ, Yoshioka M, Zender CS, et al. Change in atmospheric mineral aerosols in response to climate: Last glacial period, preindustrial, modern, and doubled carbon dioxide climates. J Geophys Res Atmos. 2006;111: D10202. doi: 10.1029/2005JD006653.

S2.1.6. Jarvis A, HI, Reuter A, Nelson A, Guevara E. Hole-filled SRTM for the globe Version 4; 2008 [cited 2020 March 5]. In: CGIAR-CSI SRTM 90m Database [Internet]. Available from: http://srtm.csi.cgiar.org.

S2.1.7. Hengl T, Mendes de Jesus J, Heuvelink GBM, Ruiperez Gonzalez M, Kilibarda M, Blagotić A. Soil-Grids250m: Global gridded soil information based on machine learning. PLOS ONE. 2017;12: e0169748. doi: 10.1371/journal.pone.0169748 PMID: 28207752.

S2.1.8. Balmino G, Vales N, Bonvalot S, Briais A. Spherical harmonic modelling to ultra-high degree of Bouguer and isostatic anomalies. J Geod. 2012;86: 499-520. doi: 10.1007/s00190-011-0533-4.

S2.1.9. Pelletier JD, Broxton PD, Hazenberg P, Zeng X, Troch PA, Niu GY. A gridded global data set of soil, intact regolith, and sedimentary deposit thicknesses for regional and global land surface modeling. J Adv Model Earth Syst. 2016;8: 41-65. doi: 10.1002/2015MS000526.

S2.1.10. Hijmans RJ, Cameron SE, Parra JL, Jones PG, Jarvis A. Very high-resolution interpolated climate surfaces for global land areas. Int J Climatol. 2005;25: 1965-1978. doi: 10.1002/joc.1276.

S2.1.11. Börker J, Hartmann J, Amann T, Romero-Mujalli G. Terrestrial sediments of the earth: development of a global unconsolidated sediments map database (gum). Geochem Geophys Geosystems. 2018;19: 997-1024. doi: 10.1002/2017GC007273.

S2.1.12. Zomer RJ, Trabucco A, Bossio DA, Verchot L V. Climate change mitigation: A spatial analysis of global land suitability for clean development mechanism afforestation and reforestation. Agric Ecosyst Environ. 2008;126: 67-80. doi: 10.1016/j.agee.2008.01.014.

S2.1.13. Arino O, Ramos Perez JJ, Kalogirou V, Bontemps S, Defourny P, Van Bogaert E. Global Land Cover Map; 2009 [cited 5 March 2020]. In: European Space Agency (ESA) & UniversiteÂ catholique de Louvain (UCL) [Internet].doi: [10.1594/PANGAEA.787668](https://doi.org/10.1594/PANGAEA.787668).

S2.1.14. Potter P, Ramankutty N, Bennett EM, Donner SD. Characterizing the spatial patterns of global fertilizer application and manure production. Earth Interact. 2010;14: 1–22. doi: 10.1175/2009EI288.1.

S2.1.15. Vet R, Artz RS, Carou S, Shaw M, Ro CU, Aas W, et al. A global assessment of precipitation chemistry and deposition of sulfur, nitrogen, sea salt, base cations, organic acids, acidity and pH, and phosphorus. Atmos Environ. 2014;93: 3-100. doi: 10.1016/j.atmosenv.2013.10.060.

S2.1.16. Newsome PF, Wilde RH, Willoughby EJ. Land Resource Information System Spatial Data Layers; 2008 [cited 5 March 2020]. In: Landcare Research New Zealand (LRIS) portal [Internet]. Available from: https://lris.scinfo.org.nz/.

S2.1.17. Leathwick J, Morgan F, Wilson G, Rutledge D, McLeod M, Johnston K. Land environments of New Zealand: a technical guide. Hamilton: Ministry for the Environment, Wellington, and Manaaki Whenua Landcare Research; 2002.

S2.1.18. GNS Science. QMap Geological Map of New Zealand 1:250,000; 2014 [cited 9 June 2020]. In: GNS Science Geological Map 1 web map application [Internet]. Available from: https://data.gns.cri.nz/geology/.

S2.1.19. Feldman GC. Distance to the Nearest Coast; 2009 [cited 1 March 2021]. In: NASA EarthData OceanColor Webmaster [Internet]. Available from: https://oceancolor.gsfc.nasa.gov/docs/distfromcoast/.
