# Supplementary material for: A bioavailable strontium (87Sr/86Sr) isoscape for Aotearoa New Zealand: Implications for food forensics and biosecurity
Source: PLoS One. 2022 Mar 16;17(3):e0264458. doi: 10.1371/journal.pone.0264458 (PMC8926269; doi:10.1371/journal.pone.0264458)
Supplement: S3 File — S3 Fig 1. Bioavailable 87Sr/86Sr isoscape (color version). The highest 87Sr/86Sr ratios are depicted in blue and the lowest are shown in purple. The bioavailable 87Sr/86Sr isoscape (R2 = 0.53, RMSE = 0.00098) demonstrates the predicted 87Sr/86Sr values, ranging from 0.70567 to 0.71118, for the entire country including the Chatham Islands. Figure developed in ArcGIS Pro using a coastlines feature layer (sourced from Natural Earth) and projected to NZTM 2000. (PDF) [file pone.0264458.s003.pdf]

S3. BIOAVAILABLE $^{87}Sr/^{86}Sr$ ISOSCAPE – COLOR VERSION

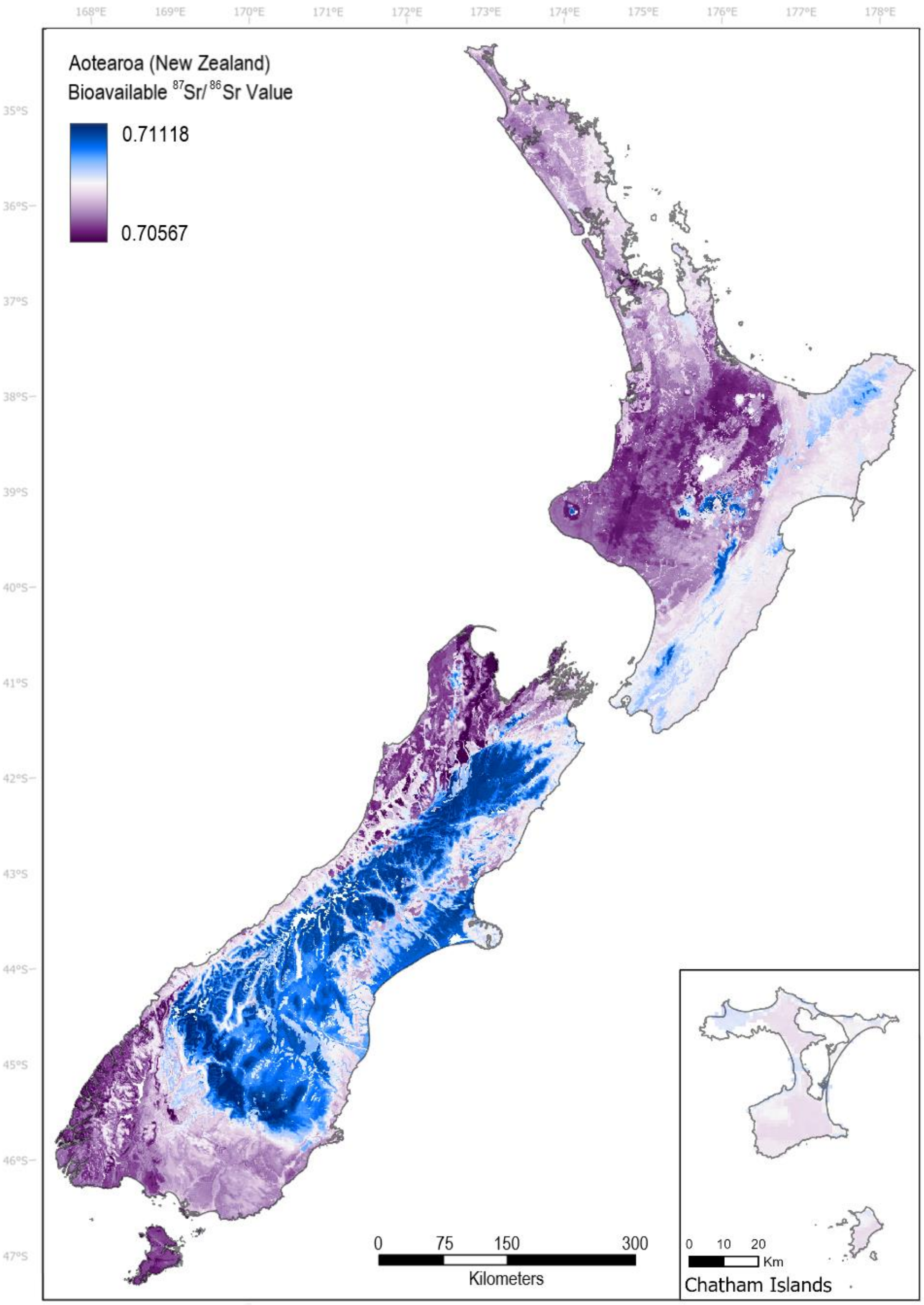

S3 Fig 1. Bioavailable $^{87}Sr/^{86}Sr$ isoscape (color version). The highest $^{87}Sr/^{86}Sr$ ratios are depicted in blue and the lowest are shown in purple. The bioavailable $^{87}Sr/^{86}Sr$ isoscape (R^2 = 0.53, RMSE = 0.00098) demonstrates the predicted $^{87}Sr/^{86}Sr$ values, ranging from 0.70567 to 0.71118, for the entire country including the Chatham Islands. Figure developed in ArcGIS Pro using a coastlines feature layer (sourced from Natural Earth) and projected to NZTM 2000.
