# Supplementary material for: A bioavailable strontium (87Sr/86Sr) isoscape for Aotearoa New Zealand: Implications for food forensics and biosecurity
Source: PLoS One. 2022 Mar 16;17(3):e0264458. doi: 10.1371/journal.pone.0264458 (PMC8926269; doi:10.1371/journal.pone.0264458)
Supplement: S5 File — PDF = “S5_CowMilkSamplePredictions”. Cow Milk Sample Predictions—Top 33% Posterior Probabilities. Regions highlighted in blue represent the top 33% of areas predicted as potential regions-of-origin using bioavailable 87Sr/86Sr where values “1” = Likely Origin and “2” = Unlikely Origin. The box in top left shows the area surrounding the cow milk sample’s actual place-of-origin marked by yellow circle. The hillshade layer was created using a GNS DEM 8m shapefile. (PDF) [file pone.0264458.s005.pdf]

## 4. COW MILK MAPS – TOP 33% POSTERIOR PROBABILITIES

Regions highlighted in blue represent the top 33% of areas predicted as potential regions-of-origin using bioavailable $^{87}Sr/^{86}Sr$ where values "1" = Likely Origin and "2" = Unlikely Origin. The box in top left shows the area surrounding the cow milk sample's actual place-of-origin marked by yellow circle. The hillshade layer was created using a GNS DEM 8m shapefile.

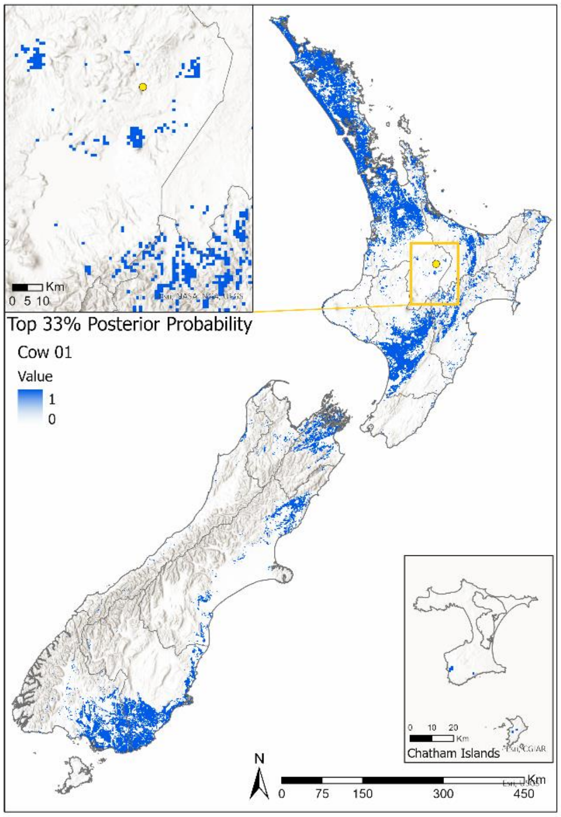


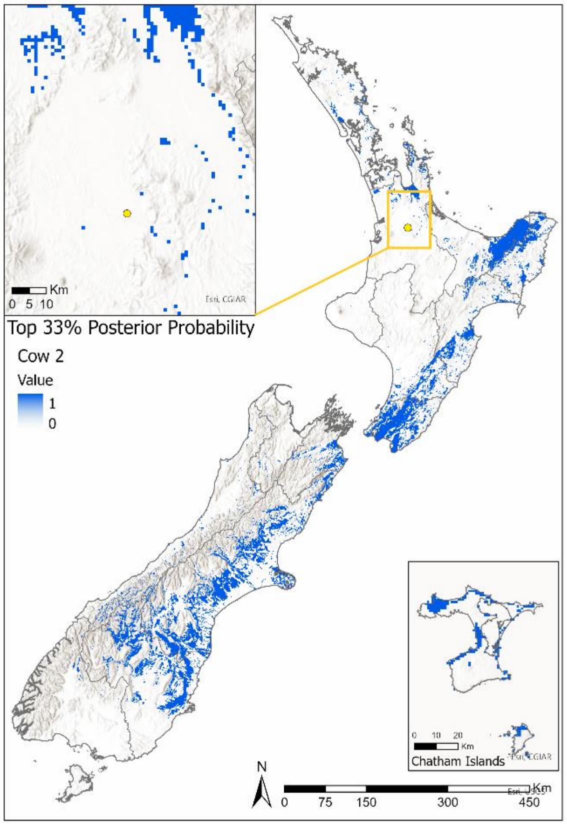


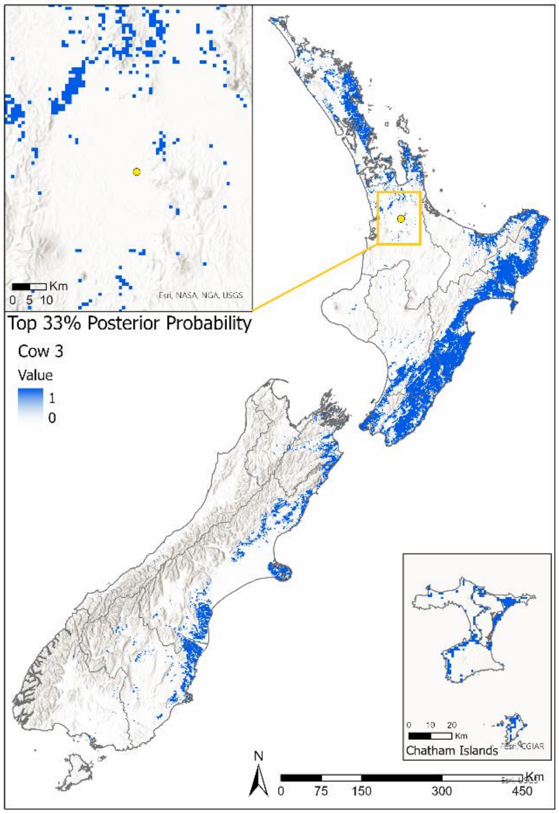


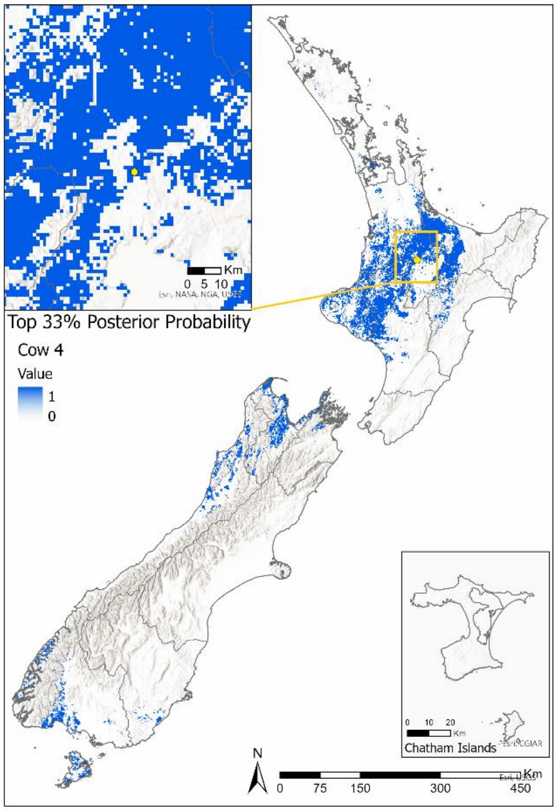

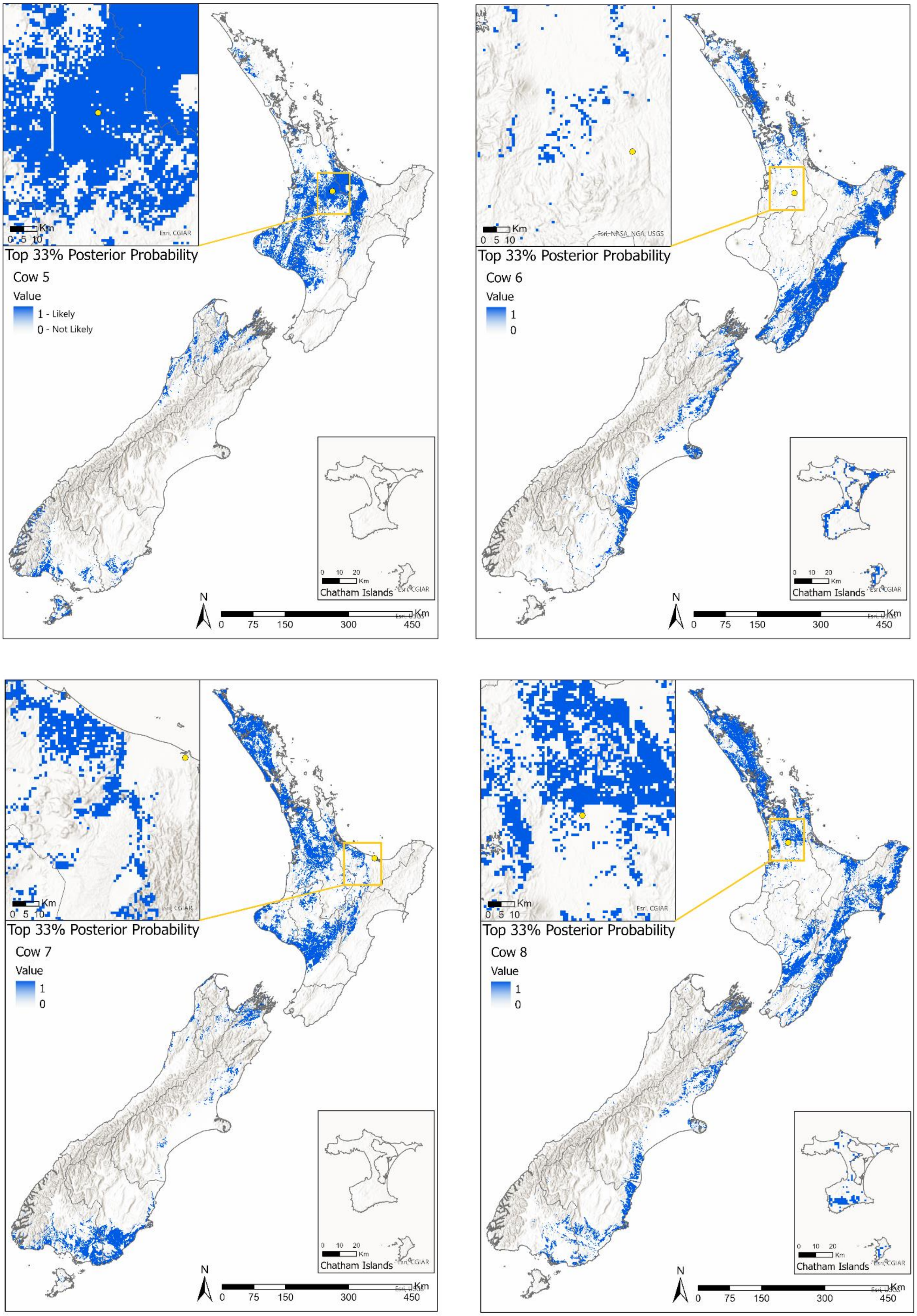

Top 33% Posterior Probability
Cow 5
Value
1 - Likely
0 - Not Likely
Chatham Islands
Esri, CGIAR
N
0 75 150 300 450 Km
Top 33% Posterior Probability
Cow 6
Value
1
0
Esri, NASA, NGA, USGS
Chatham Islands
N
0 75 150 300 450 Km
Top 33% Posterior Probability
Cow 7
Value
1
0
Chatham Islands
N
0 75 150 300 450 Km
Top 33% Posterior Probability
Cow 8
Value
1
0
Chatham Islands
N
0 75 150 300 450 Km

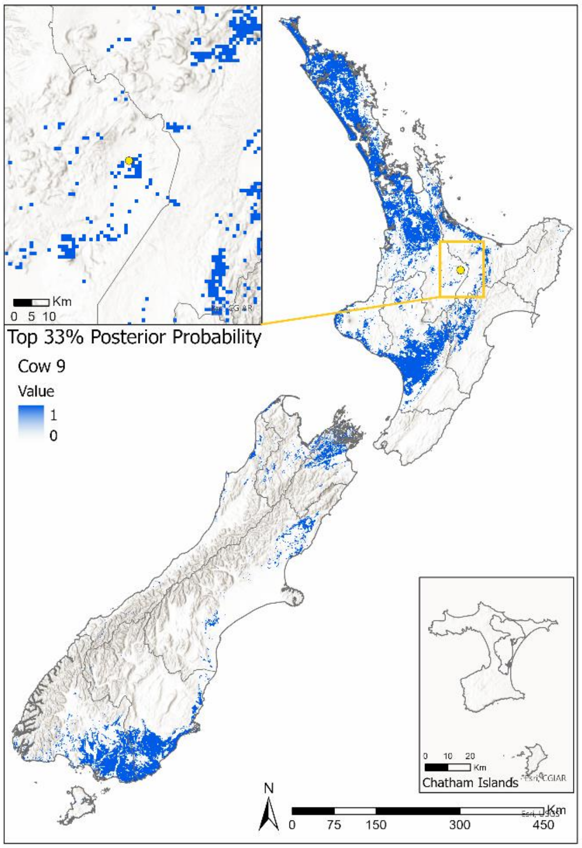
Km
0 5 10
Top 33% Posterior Probability
Cow 9
Value
1
0
0 10 20
Km
Chatham Islands
N
Km
0 75 150 300 450

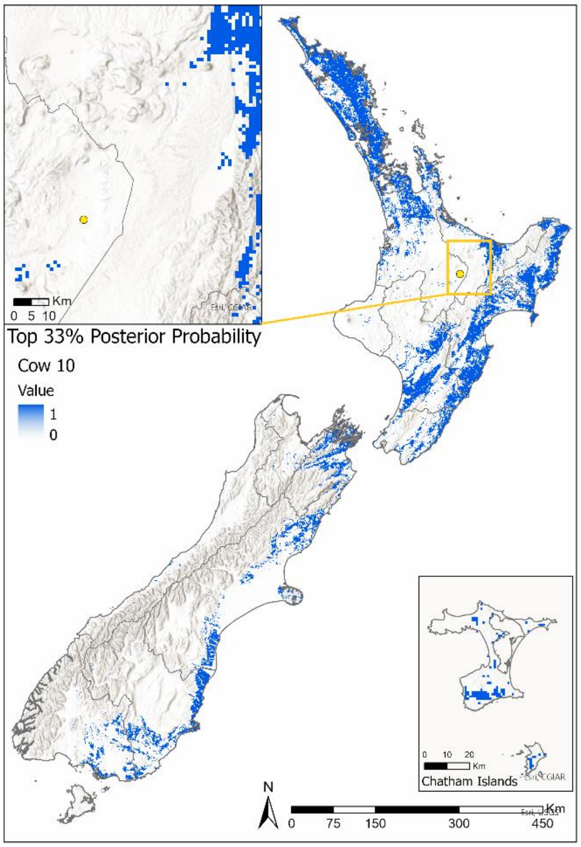
Km
0 5 10
Top 33% Posterior Probability
Cow 10
Value
1
0
0 10 20
Km
Chatham Islands
N
Km
0 75 150 300 450

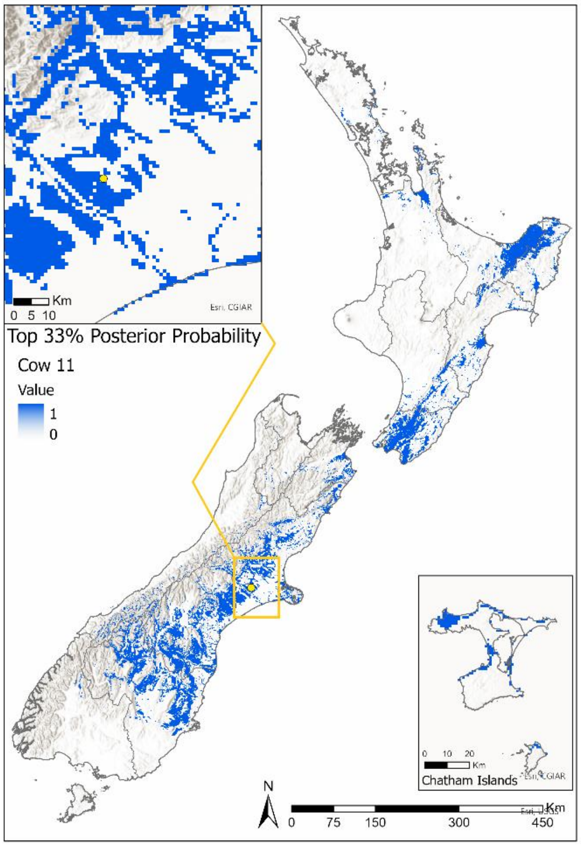
Km
0 5 10
Esri, CGIAR
Top 33% Posterior Probability
Cow 11
Value
1
0
0 10 20
Km
Chatham Islands
N
Km
0 75 150 300 450

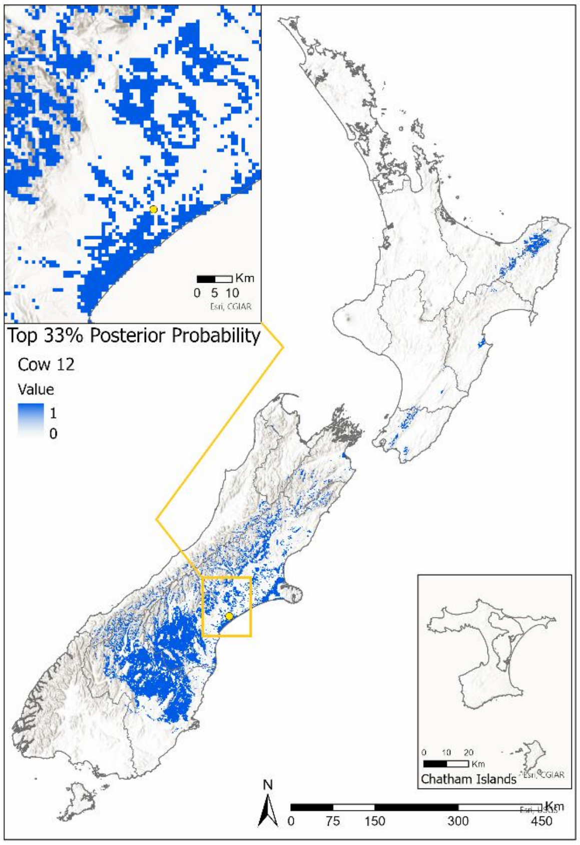
Km
0 5 10
Esri, CGIAR
Top 33% Posterior Probability
Cow 12
Value
1
0
0 10 20
Km
Chatham Islands
N
Km
0 75 150 300 450

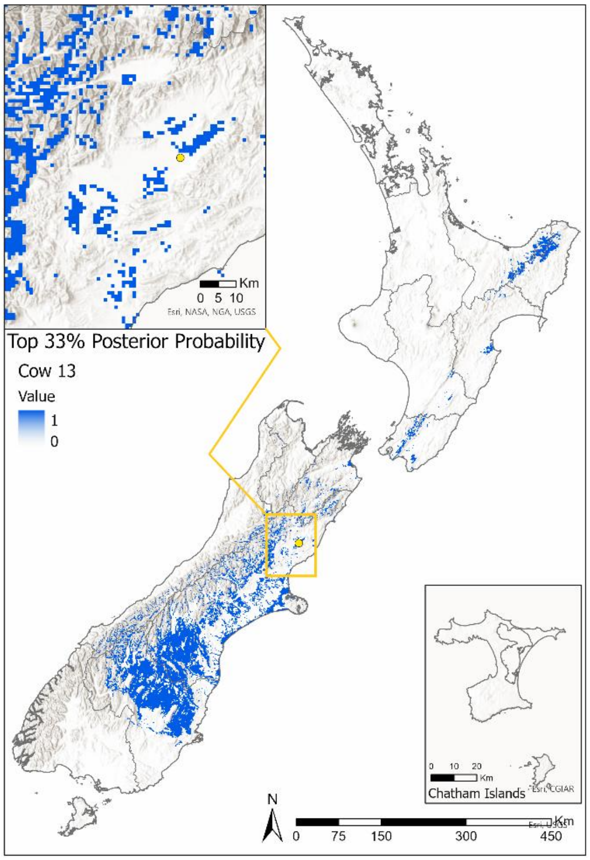
Km
0 5 10
Esri, NASA, NGA, USGS
Top 33% Posterior Probability
Cow 13
Value
1
0
0 10 20
Km
Chatham Islands
Esri, CGIAR
N
Km
0 75 150 300 450
Esri, USGS

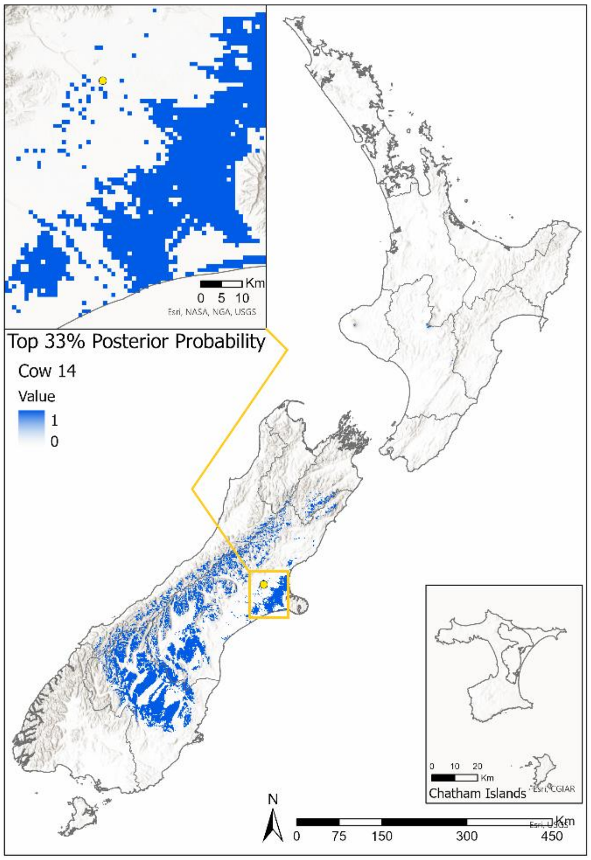
Km
0 5 10
Esri, NASA, NGA, USGS
Top 33% Posterior Probability
Cow 14
Value
1
0
0 10 20
Km
Chatham Islands
Esri, CGIAR
N
Km
0 75 150 300 450
Esri, USGS

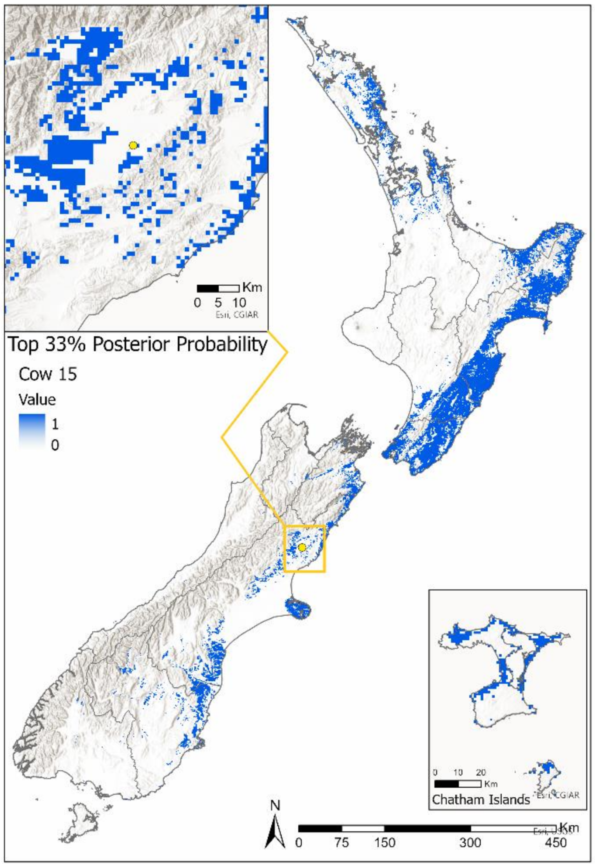
Km
0 5 10
Esri, CGIAR
Top 33% Posterior Probability
Cow 15
Value
1
0
0 10 20
Km
Chatham Islands
Esri, CGIAR
N
Km
0 75 150 300 450

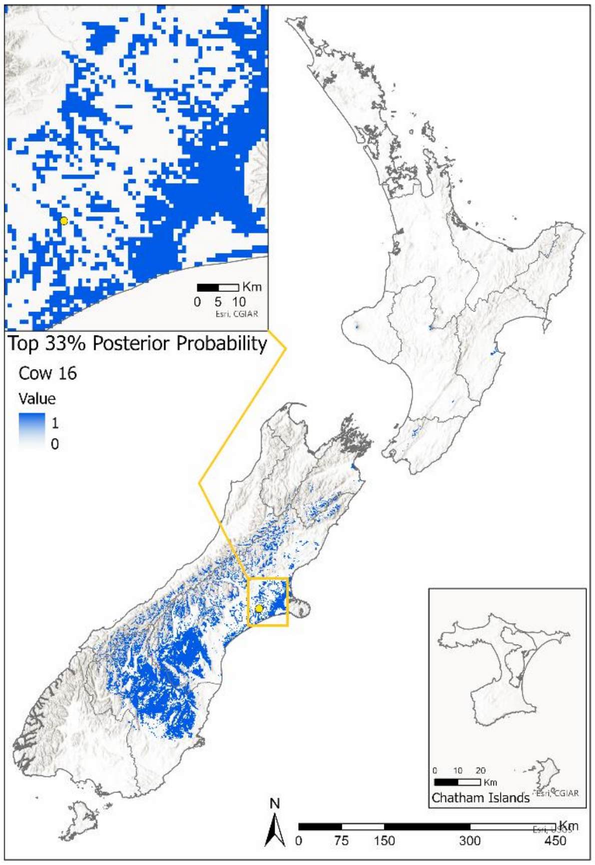
Km
0 5 10
Esri, CGIAR
Top 33% Posterior Probability
Cow 16
Value
1
0
0 10 20
Km
Chatham Islands
Esri, CGIAR
N
Km
0 75 150 300 450

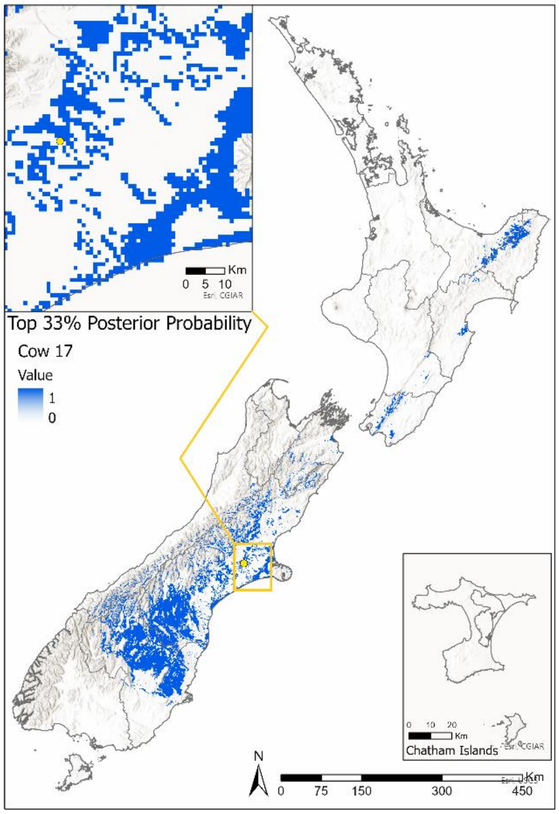

Top 33% Posterior Probability
Cow 17
Value
1
0
Km
0 5 10
Esri, CGIAR
Chatham Islands
N
Km
0 75 150 300 450


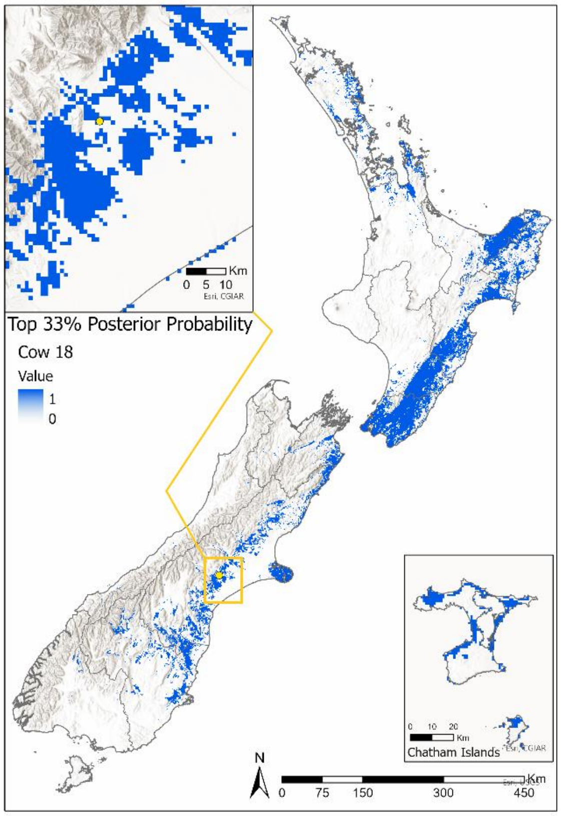

Top 33% Posterior Probability
Cow 18
Value
1
0
Km
0 5 10
Esri, CGIAR
Chatham Islands
N
Km
0 75 150 300 450


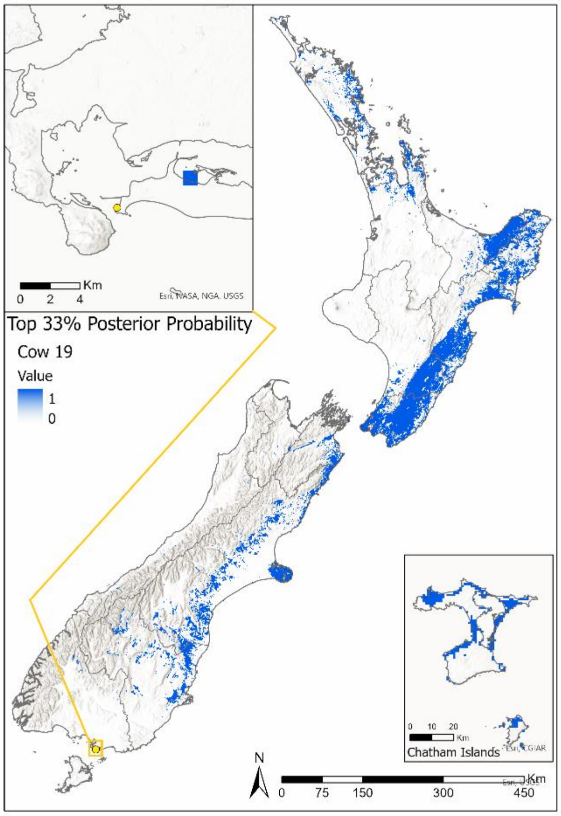

Top 33% Posterior Probability
Cow 19
Value
1
0
Km
0 2 4
Esri, NASA, NGA, USGS
Chatham Islands
N
Km
0 75 150 300 450


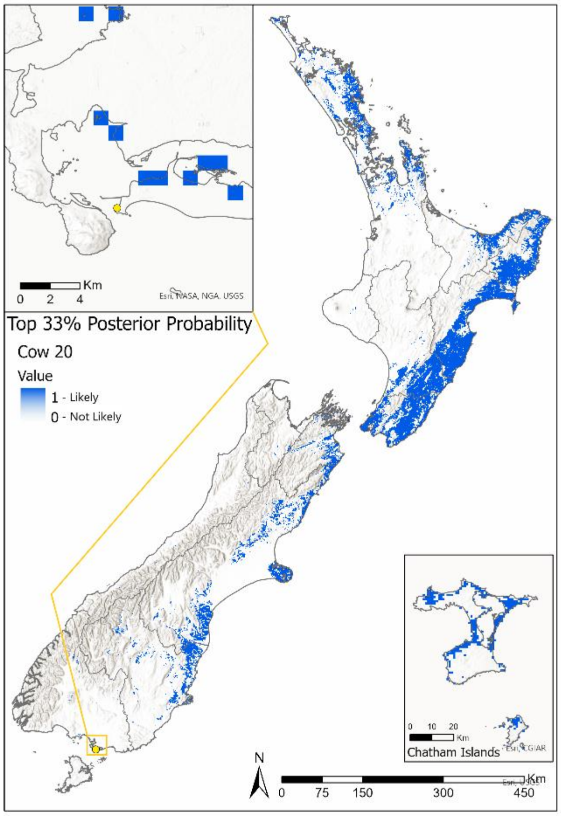

Top 33% Posterior Probability
Cow 20
Value
1 - Likely
0 - Not Likely
Km
0 2 4
Esri, NASA, NGA, USGS
Chatham Islands
N
Km
0 75 150 300 450

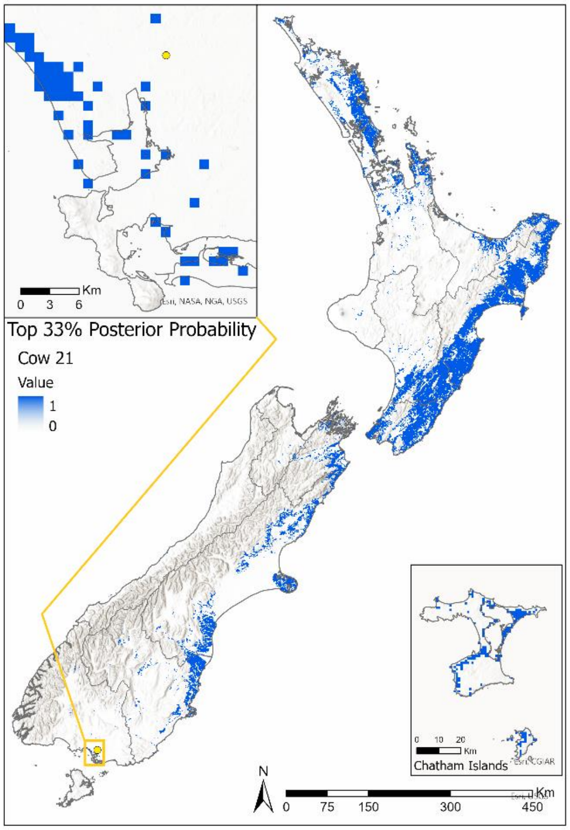
Top 33% Posterior Probability
Cow 21
Value
1
0
Km
0 3 6
Esri, NASA, NGA, USGS
Chatham Islands
0 75 150 300 450
Km
N

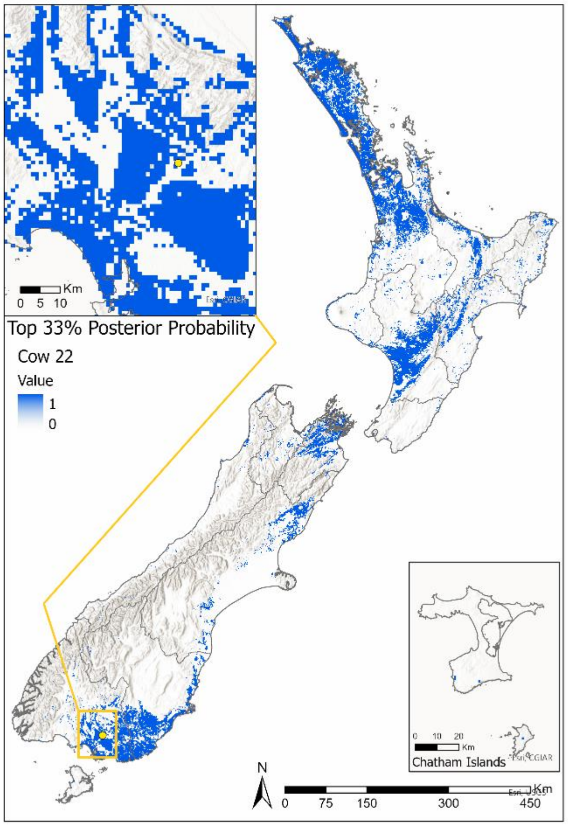
Top 33% Posterior Probability
Cow 22
Value
1
0
Km
0 5 10
Chatham Islands
0 75 150 300 450
Km
N

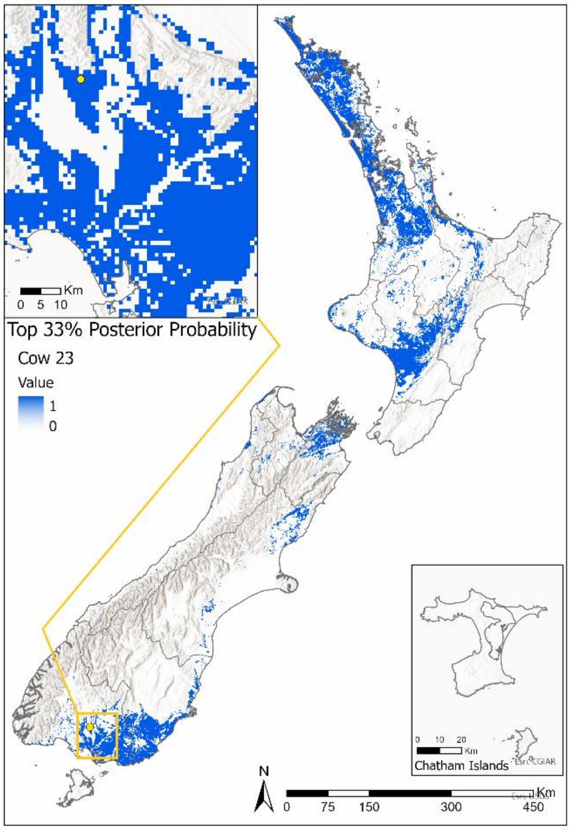
Top 33% Posterior Probability
Cow 23
Value
1
0
Km
0 5 10
Chatham Islands
0 75 150 300 450
Km
N

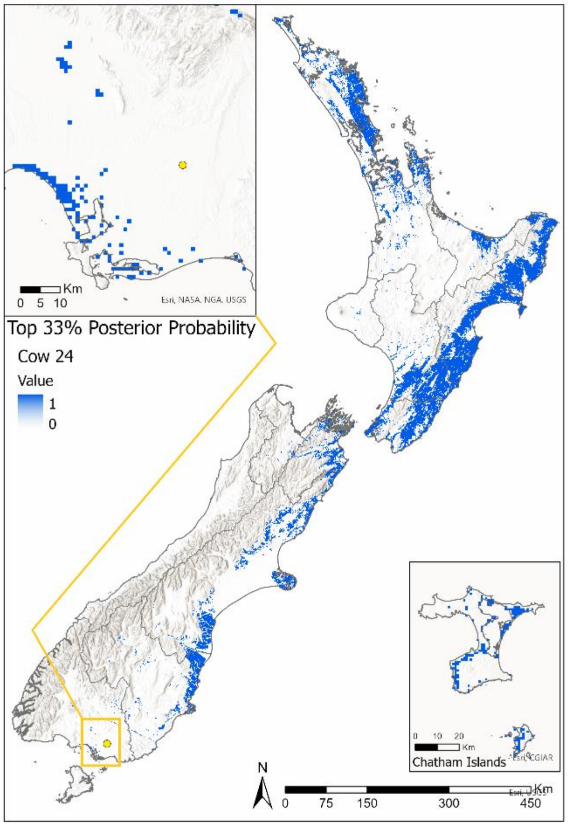
Top 33% Posterior Probability
Cow 24
Value
1
0
Km
0 5 10
Esri, NASA, NGA, USGS
Chatham Islands
0 75 150 300 450
Km
N

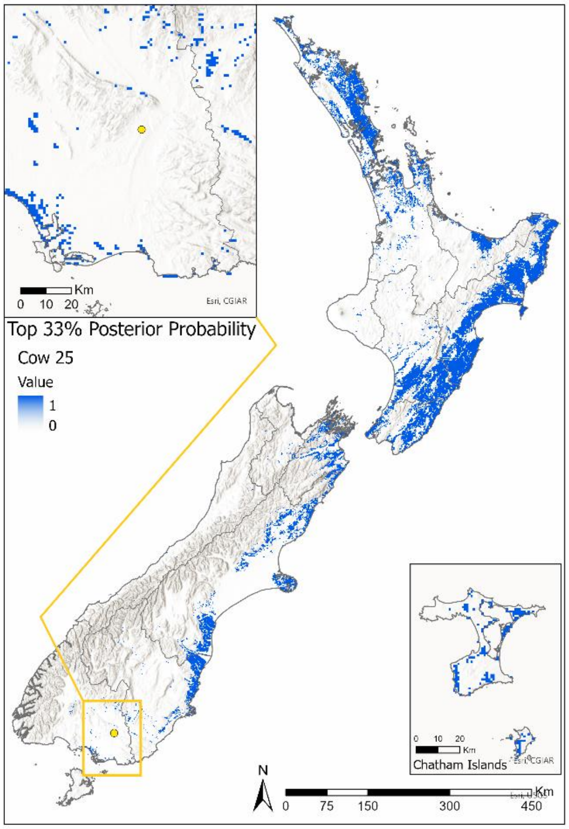
Km
0 10 20
Esri, CGIAR
Top 33% Posterior Probability
Cow 25
Value
1
0
Chatham Islands
N
0 75 150 300 450
Km

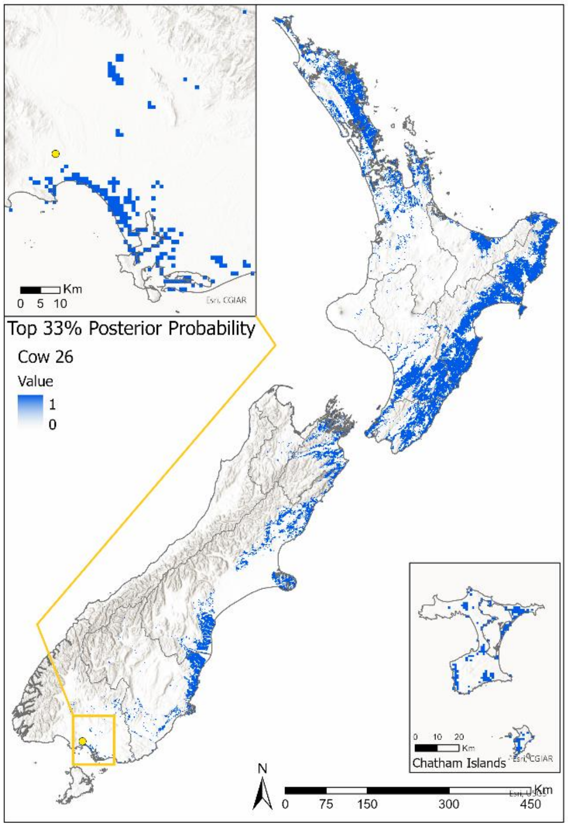
Km
0 5 10
Esri, CGIAR
Top 33% Posterior Probability
Cow 26
Value
1
0
Chatham Islands
N
0 75 150 300 450
Km

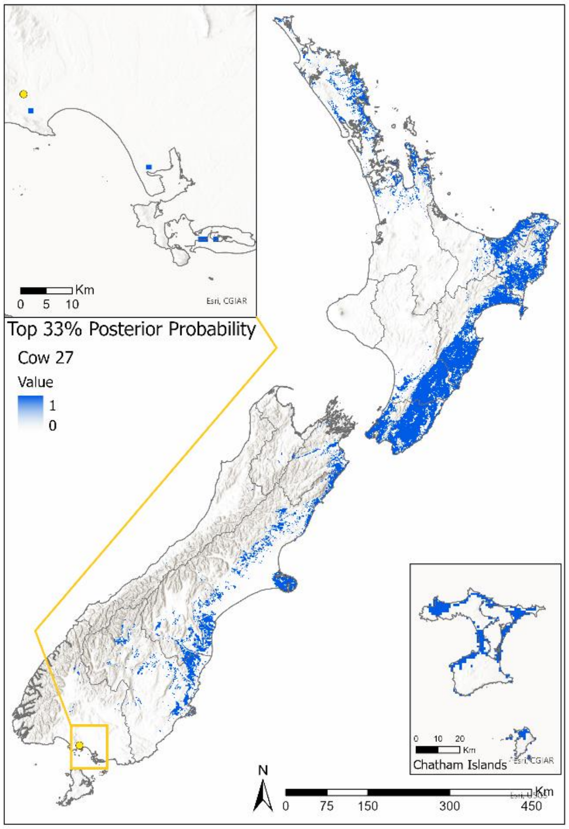
Km
0 5 10
Esri, CGIAR
Top 33% Posterior Probability
Cow 27
Value
1
0
Chatham Islands
N
0 75 150 300 450
Km

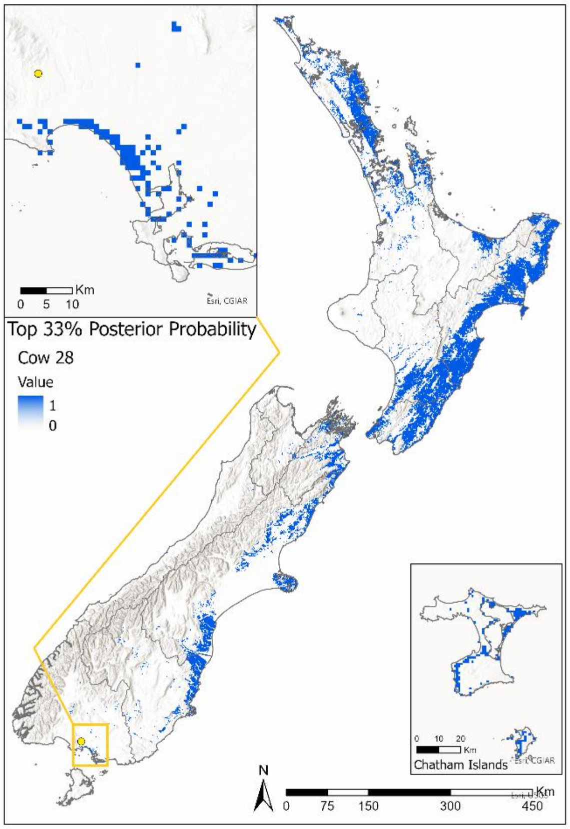
Km
0 5 10
Esri, CGIAR
Top 33% Posterior Probability
Cow 28
Value
1
0
Chatham Islands
N
0 75 150 300 450
Km

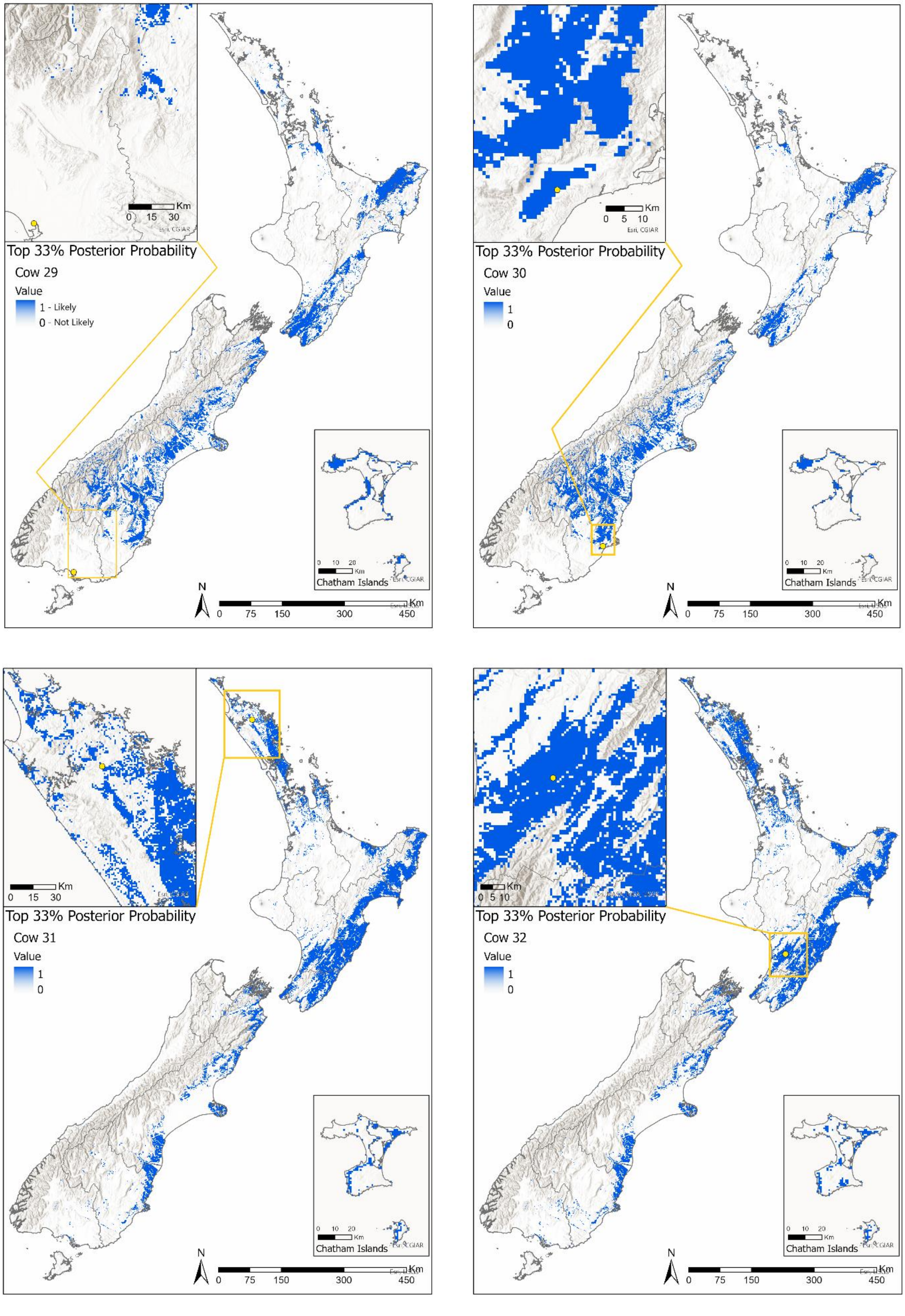
Top 33% Posterior Probability
Cow 29
Value
1 - Likely
0 - Not Likely
Km
0 15 30
Esri, CGIAR
Chatham Islands
N
Km
0 75 150 300 450
Top 33% Posterior Probability
Cow 30
Value
1
0
Km
0 5 10
Esri, CGIAR
Chatham Islands
Top 33% Posterior Probability
Cow 31
Value
1
0
Km
0 15 30
Chatham Islands
Top 33% Posterior Probability
Cow 32
Value
1
0
Km
0 5 10
Chatham Islands

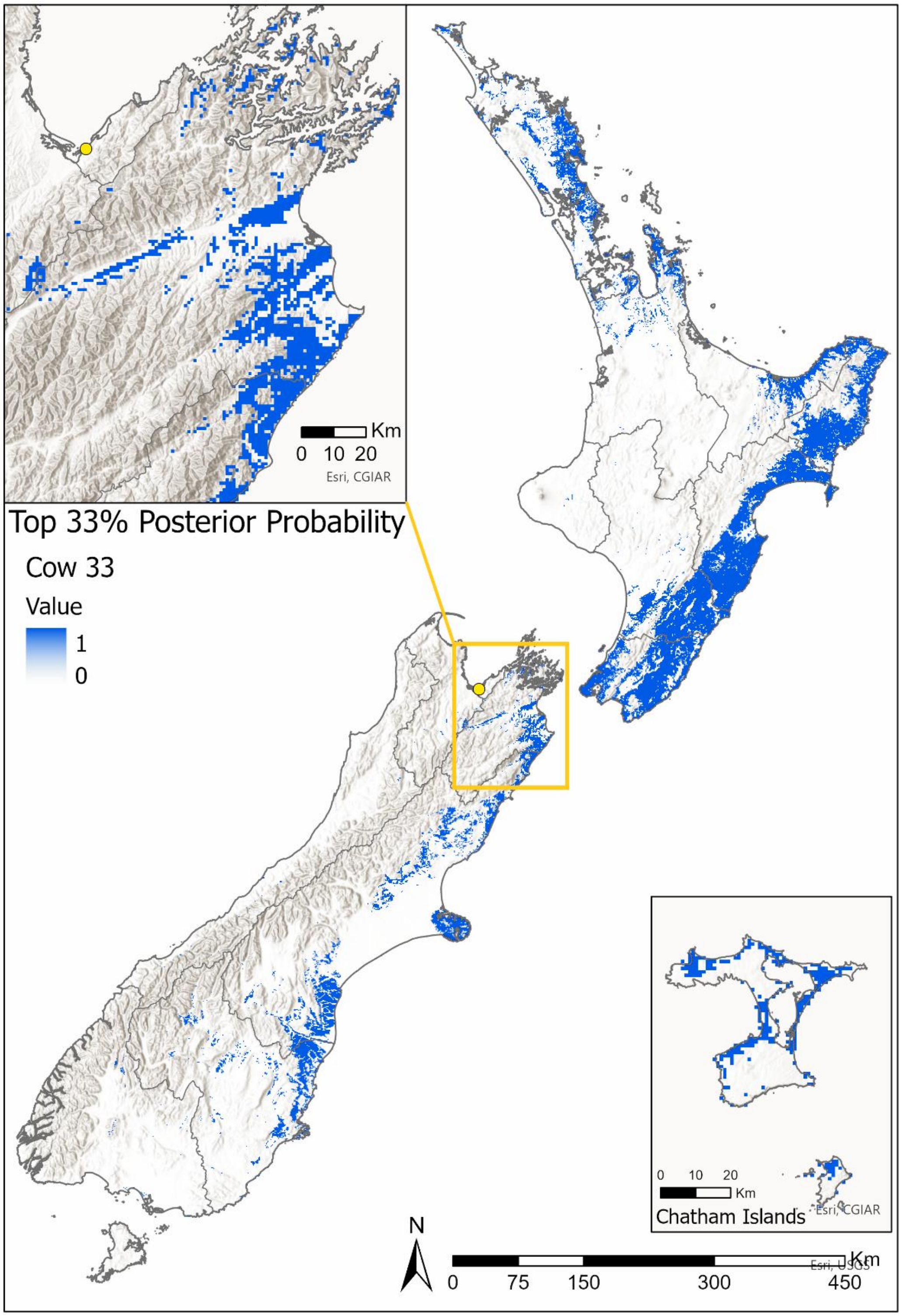

Km
0 10 20
Esri, CGIAR
Top 33% Posterior Probability
Cow 33
Value
1
0
0 10 20
Km
Chatham Islands
Esri, CGIAR
N
Km
0 75 150 300 450
Esri, USGS
