# Supplementary material for: A bioavailable strontium (87Sr/86Sr) isoscape for Aotearoa New Zealand: Implications for food forensics and biosecurity
Source: PLoS One. 2022 Mar 16;17(3):e0264458. doi: 10.1371/journal.pone.0264458 (PMC8926269; doi:10.1371/journal.pone.0264458)
Supplement: S6 File — (DOCX) [file pone.0264458.s006.docx]

**S6. REFERENCES**

S5.1. TRACE. Standard Operating Procedures: Soil and water sampling. 2005; 1-20.

S5.2. McArthur JM. Recent trends in strontium isotope stratigraphy. Terra Nova. 1994;6: 331-58. doi: 10.1111/j.1365-3121.1994.tb00507.x.

S5.3. Ayyadevara VK. Pro Machine Learning Algorithms: A Hands-On Approach to Implementing Algorithms in Python and R. Berkeley: Apress; 2018. doi: 10.1007/978-1-4842-3564-5.

S5.4. Awad M, Khanna R. Efficient Learning Machines: theories, concepts, and applications for engineers and system designers. Springer Nature: New York; 2015.

S5.5. Vaysse K, Lagacherie P. Using quantile regression forest to estimate uncertainty of digital soil mapping products. Geoderma. 2017;291: 55-64. doi: 10.1016/j.geoderma.2016.12.017.

S5.6. Mooney WD, Laske G, Masters TG. CRUST 5.1: A global crustal model at 5Ê × 5Ê. J Geophys Res Solid Earth. 1998;103: 727-747. doi: 10.1029/97JB02122.

S5.7. Mahowald NM, Muhs DR, Levis S, Rasch PJ, Yoshioka M, Zender CS, et al. Change in atmospheric mineral aerosols in response to climate: Last glacial period, preindustrial, modern, and doubled carbon dioxide climates. J Geophys Res Atmos. 2006;111: D10202. doi: 10.1029/2005JD006653.

S5.8. Jarvis A, HI, Reuter A, Nelson A, Guevara E. Hole-filled SRTM for the globe Version 4; 2008 [cited 2020 March 5]. In: CGIAR-CSI SRTM 90m Database [Internet]. Available from: http://srtm.csi.cgiar.org.

S5.9. Hengl T, Mendes de Jesus J, Heuvelink GBM, Ruiperez Gonzalez M, Kilibarda M, Blagotić A. Soil-Grids250m: Global gridded soil information based on machine learning. PLOS ONE. 2017;12: e0169748. doi: 10.1371/journal.pone.0169748 PMID: 28207752.

S5.10. Balmino G, Vales N, Bonvalot S, Briais A. Spherical harmonic modelling to ultra-high degree of Bouguer and isostatic anomalies. J Geod. 2012;86: 499-520. doi: 10.1007/s00190-011-0533-4.

S5.11. Pelletier JD, Broxton PD, Hazenberg P, Zeng X, Troch PA, Niu GY. A gridded global data set of soil, intact regolith, and sedimentary deposit thicknesses for regional and global land surface modeling. J Adv Model Earth Syst. 2016;8: 41-65. doi: 10.1002/2015MS000526.

S5.12. Hijmans RJ, Cameron SE, Parra JL, Jones PG, Jarvis A. Very high-resolution interpolated climate surfaces for global land areas. Int J Climatol. 2005;25: 1965-1978. doi: 10.1002/joc.1276.

S5.13. Börker J, Hartmann J, Amann T, Romero-Mujalli G. Terrestrial sediments of the earth: development of a global unconsolidated sediments map database (gum). Geochem Geophys Geosystems. 2018;19: 997-1024. doi: 10.1002/2017GC007273.

S5.14. Zomer RJ, Trabucco A, Bossio DA, Verchot L V. Climate change mitigation: A spatial analysis of global land suitability for clean development mechanism afforestation and reforestation. Agric Ecosyst Environ. 2008;126: 67-80. doi: 10.1016/j.agee.2008.01.014.

S5.15. Arino O, Ramos Perez JJ, Kalogirou V, Bontemps S, Defourny P, Van Bogaert E. Global Land Cover Map; 2009 [cited 5 March 2020]. In: European Space Agency (ESA) & UniversiteÂ catholique de Louvain (UCL) [Internet].doi: [10.1594/PANGAEA.787668](https://doi.org/10.1594/PANGAEA.787668).

S5.16. Potter P, Ramankutty N, Bennett EM, Donner SD. Characterizing the spatial patterns of global fertilizer application and manure production. Earth Interact. 2010;14: 1–22. doi: 10.1175/2009EI288.1.

S5.17. Vet R, Artz RS, Carou S, Shaw M, Ro CU, Aas W, et al. A global assessment of precipitation chemistry and deposition of sulfur, nitrogen, sea salt, base cations, organic acids, acidity and pH, and phosphorus. Atmos Environ. 2014;93: 3-100. doi: 10.1016/j.atmosenv.2013.10.060.

S5.18. Newsome PF, Wilde RH, Willoughby EJ. Land Resource Information System Spatial Data Layers; 2008 [cited 5 March 2020]. In: Landcare Research New Zealand (LRIS) portal [Internet]. Available from: https://lris.scinfo.org.nz/.

S5.19. Leathwick J, Morgan F, Wilson G, Rutledge D, McLeod M, Johnston K. Land environments of New Zealand: a technical guide. Hamilton: Ministry for the Environment, Wellington, and Manaaki Whenua Landcare Research; 2002.

S5.20. GNS Science. QMap Geological Map of New Zealand 1:250,000; 2014 [cited 9 June 2020]. In: GNS Science Geological Map 1 web map application [Internet]. Available from: https://data.gns.cri.nz/geology/.

S5.21. Feldman GC. Distance to the Nearest Coast; 2009 [cited 1 March 2021]. In: NASA EarthData OceanColor Webmaster [Internet]. Available from: https://oceancolor.gsfc.nasa.gov/docs/distfromcoast/.
