# Supplementary material for: A bioavailable strontium (87Sr/86Sr) isoscape for Aotearoa New Zealand: Implications for food forensics and biosecurity
Source: PLoS One. 2022 Mar 16;17(3):e0264458. doi: 10.1371/journal.pone.0264458 (PMC8926269; doi:10.1371/journal.pone.0264458)
Supplement: Attachment — Submitted filename: Response to Reviewers.docx [file pone.0264458.s007.docx]

Dear Lee and Reviewers,

Thank you all for your edits and comments. I and my co-authors greatly appreciate your feedback and have made the requested changes enumerated below. We have ensured that the manuscript meets PLOS ONE’s style requirements.

Best,

Robyn T. Kramer

**Editor’s Comments**

1. Please ensure that your manuscript meets PLOS ONE's style requirements, including those for file naming.

We have ensured that the manuscript meets PLOS ONE’s style requirements.

2. In your Methods section, please provide additional information regarding the permits you obtained to collect samples for the present study. Please ensure you have included the full name of the authority that approved the field site access and, if no permits were required, a brief statement explaining why.

Statements regarding permits and access to samples have been added to each plant and soil sample section in the Supplemental Information.

3. We note that Figures 1, 3, 7 and S1 in your submission contain [map/satellite] images which may be copyrighted. All PLOS content is published under the Creative Commons Attribution License (CC BY 4.0), which means that the manuscript, images, and Supporting Information files will be freely available online, and any third party is permitted to access, download, copy, distribute, and use these materials in any way, even commercially, with proper attribution. For these reasons, we cannot publish previously copyrighted maps or satellite images created using proprietary data, such as Google software (Google Maps, Street View, and Earth). For more information, see our copyright guidelines: <http://journals.plos.org/plosone/s/licenses-and-copyright>.

All data used in this manuscript were created specifically for this research using data produced by the authors or public domain vectors that are free of copyrights. Figures 1, 3, 7, and S1 figures were all created by the first author RTK using data produced through this research or provided by co-authors who hold the rights to the material. All figures use public-domain country outlines obtained from NaturalEarthData.com and figure captions cite where the vectors were obtained as specified in NaturalEarth’s Terms of Use (https://www.naturalearthdata.com/about/terms-of-use/). The multi-directional hill-shade layer in Fig 1 and the S1 figures was created by RTK using 8m DEM files provided by co-authors (Giovanni Pradel, Adam Martin, etc.) working for GNS Science and proper citation of this is provided in each figure using the hill-shade layer. In Fig 3, no copyrighted layers are used to construct the figure –the isoscape raster and interquartile range raster were created by RTK through this research. The strontium isoscape raster in Fig 7 was created by RTK and the terranes graphic was created by APM using his own data.

4. Please review your reference list to ensure that it is complete and correct. If you have cited papers that have been retracted, please include the rationale for doing so in the manuscript text, or remove these references and replace them with relevant current references. Any changes to the reference list should be mentioned in the rebuttal letter that accompanies your revised manuscript. If you need to cite a retracted article, indicate the article’s retracted status in the References list and also include a citation and full reference for the retraction notice.

The reference list has been reviewed.

**Review Comments to the Author**
Reviewer #1: This is a very well written manuscript and very well performed study. I have only a handful of minor comments:

1. Line 59: the "18O" font looks strange.

Change made.

2. Lines 526-528: What is the mechanism by which temperature influences bioavailable Sr isotopes?

Reworded sentence to explain that temperature corresponds with other predictor variables (elevation and geology) that do affect Sr variation.

3. The word "data" is plural is "becomes" should be "become".

Change made.

Reviewer #2: The authors provide a very well done and much needed contribution to the strontium isotope global literature in the form of a predicted isoscape with validation from Aotearoa. The production of the isoscape is rigorous and follows known best practices in the literature. Most of my comments are relatively minor. My one major question comes with the applicability sourcing using the 33% predictive threshold; while most milk samples did have a nearby highly probably source, the highest density of probable source locations was often on the other side of the country (ex: Cow 19-21)! How can this predictive model be used in a way that is meaningful to unknown samples? I go into more detail on these question in the line-by-line comments below. Overall, this is a very strong paper and a dataset worthy of publication. I thank the authors for their diligence and look forward to their revisions.


Line 32: “Bioavailable stable and radiogenic…” – This should just read “radiogenic.” There are no stable isotopes of strontium.

Change made.

Also, the word ‘ubiquitous’ feels strange here, and I’m not sure what meaning the authors are trying to convey with it. Consider selecting a different word.

STET – ubiquitous is defined as ‘found everywhere or pervasive’ and myself and my co-authors feel that it is used appropriately in the sentence.

Lines 52-68: This goes fairly in-depth right off the bat about using isotopes to track the origins of pest insects themselves; while interesting, this is not actually relevant to the focus of the paper, which tests the validity of the isoscape to source products. The discussion of the application to identifying the imported vs “homegrown” nature of pest insects is fine, but belongs in the Discussion section and not as the opening to the paper. You could also move the discussion of the isotopic application into the ‘previous research’ summary beginning on line 82.

The section was moved to the Discussion section as suggested. Thank you for the recommendation. I do think it flows a bit better now.

111 - Hydrogen and oxygen are used in many places to determine provenance within a regional scale. This has even been done with milk (Vieira Silva et al 2013, Boito et al 2021, links to journals below). The authors are not incorrect about the ambiguity surrounding all the inputs into an oxygen isotope ratio, but that does not mean that it cannot be used at a regional scale. Like strontium, it all depends on the variation present and the patterning of that variation!

I explained further and changed previous sentence to say: “Generally, however, all else being equal it can be challenging to isotopically distinguish between ecosystems that are geologically and climatically similar using only δ^2^H and δ^18^O [40,42] because the variation and patterning of atmospheric isotopes may not be distinct enough. This issue is most important in Aotearoa and the greater Pacific where islands and small land masses do not experience the same continentality effects that lead to distinct isotopic variation in other regions of the world.”

129-131 – isn’t this same limitation true of strontium isotope ratios? Without baseline knowledge – something your isoscape is providing here! – you are unable to draw conclusions about provenance.

STET – Yes, but strontium isotopes can be predicted for non-sampled areas, while (typically) trace elements do not use predicted values. They are limited to a direct comparison between the unknown sample against the known sample.

172 – Isoscapes for Tanzania and Kenya should be included here as well (Janzen et al 2020).

Citation added. Thank you for the suggestion!

199 – Riparian areas are also shown to influence the values of plants growing near mobile waterways because of the variable values from that water flowing over different isotopic zones (Sillen et al 1998, Hamilton et al 2019). This should also be included in the discussion of possible influences on the isotopic ratio, and discussed in the context of the authors’ collection of samples (were any samples in riparian zones? Could this influence results?)

Riparian areas were avoided for this reason. Following sentence was added to the methods section explaining sampling protocol: Riparian areas, land in flood areas and along watercourses, were avoided to avoid any mixing of exogenous Sr sources [98].

206 – were samples collected far enough from roads to avoid contamination from dust from vehicles, etc? Also, include information about topical cleaning of samples (were they brushed clean or washed before being dehydrated, for example) or other precautions taken to avoid dust contamination in the measurement.

Dust and other potential contaminants were removed from samples by leaching the plant leaves in 2% HNO3 acid for 15 minutes and then rinsed three times with milliQ water before being digested in the microwave. This detailed information is provided in the Supplemental Information for plants collected for this study.

290 – what is the turnover time for milk production? In other words, how long would cows need to be feeding locally to ensure that the signal within the milk produced is local?

We were not able to find any dietary studies that answered this question which is one of the reasons that Kavi Wijenayake requested cow milk samples from cows that were only allowed to eat local grass diets with no supplementary feed added.

368 – how can the categorical variable of “principal surface lithology type” be inversely correlated with something? How can a categorical variable increase or decrease?

A line was added stating, “Because this variable is categorical, its correlation with ^87^Sr/^86^Sr is most likely coincidental.”

Line 400 / Fig 6 – Can you explicitly discuss the source the predicted Sr/Sr value in the paper body? You describe it well in the figure 6 caption as being based on the isoscape and known area of sample collection, but the way it reads in the paper makes it sound like it is a value related to the posterior probabily map somehow.

Added “from the isoscape” or changed wording to “predicted ^87^Sr/^86^Sr isoscape values” when appropriate to help clarify that the predicted values were obtained from the ^87^Sr/^86^Sr isoscape.


404 – Line 404 – remove comma after “all”

Change made.

Here is my major question with this particular study. I appreciate the methodology used here, and the assignR function is an excellent analytical choice. However, looking at the provided maps, I have no idea how this information would be useful for an unknown sample in providing provenience information. The metric of “distance to the nearest predicted origin” feels somewhat misleading, given how many high-probability cells there are all over the map, often at much greater densities far away from the true point of origin. Given this, how would this method actually be useful to determining the validity of a product’s stated origin? You discuss that the accuracy goes down while precision goes up using smaller threshholds, but isn’t that exactly what you would expect? Without the precision, how is this technique useful in practice?

The predictions do behave as expected, where the larger the threshold is, the more accurate and less precise the predictions are and vice versa. In this paper, we wanted to establish that the Aotearoa ^87^Sr/^86^Sr isoscape can predict the region-of-origin using the assignR method. Also, we acknowledge that predictions based solely on ^87^Sr/^86^Sr isotopes are accurate but will lack precision. In the concluding statement, we state that geological isotopes, like ^87^Sr/^86^Sr, should be included alongside atmospheric isotopes, like ō^2^H and ō^18^O, to bolster the predictive strength of future provenancing applications. Specifically, we state that “Future provenancing projects in Aotearoa should implement a multi-isotope approach that uses ^87^Sr/^86^Sr alongside other light stable isotopes to produce both accurate and precise region-of-origin predictions”.

We use the distance measurements and the top 33% quantile technique as a way to standardize prediction outputs and to quantify/assess the accuracy of predictions. Without the distance measurements, we could only say whether or not the region-of-origin was highlighted. The distance measurement allowed us to have a quantitative assessment of the accuracy for each prediction.

(I am working on another manuscript that performs dual-isotope, ^87^Sr/^86^Sr and ō^18^O, assignments for archaeological human remains in Aotearoa and the predictions using the top 33% quantile are far more precise than using only ^87^Sr/^86^Sr, as expected. That paper specifically compares the precision of ^87^Sr/^86^Sr-only predictions versus ^87^Sr/^86^Sr and ō^18^O predictions. Including those findings in this manuscript made the paper far too long and complicated.)

427 – Is the accuracy/precision of estimates related to the general heterogeneity of the area? I would image the more heterogenous (geologically) the area is, the more precise the estimate might be, whereas more homogenous areas might have higher accuracy with less precision. Do you see any correlations like this?

Partly, we found that the predictions were most accurate in regions that exhibited either very high or very low ^87^Sr/^86^Sr values. Geologically homogenous regions, like the Canterbury Plains and the eastern coast of the North Island, were highlighted as potential origins for many samples. When these regions were predicted, they tended to produce very imprecise probability surfaces that highlighted massive regions of the map that were geologically similar to one another. Because more regions were highlighted, the accuracies of these samples tended to be high. However, this seems like a false positive because, while the accuracy may be high, if you have no a priori information about the sample, it will be nearly impossible to infer origin without the addition of another isotope system or another line of evidence in general.

526 and 590 – Is there any proposal causal mechanism in the literature to explain MAT impacting strontium ratio? I have never seen this proposed, and can’t think of a reason why temperature alone would change the isotopic composition of an area. It reads here as causal (MAT causes Sr/Sr to change) – is that accurate, or is it merely a correlation observed (because of interaction effects with elevation, age, lithology, etc)? Please be clear.

Exactly as you suggest. I believe that MAT is simply correlated with other predictor variables like elevation and lithology that do have an effect on bioavailable ^87^Sr/^86^Sr values. In response to Reviewer #1 comments, I added the following to the manuscript: “The mean annual temperature plot (Fig 5) demonstrates that temperature has a significant effect on bioavailable ^87^Sr/^86^Sr in Aotearoa where higher ^87^Sr/^86^Sr values occur in areas with lower temperatures. This pattern correlates with geological and latitudinal differences between the North and South Islands. The former experiences warmer weather and has more volcanic and low-Sr lithologies (Fig 7), while the latter experiences colder weather and the highest ^87^Sr/^86^Sr values occur along the Southern Alps and the Canterbury plains.”

620 – this would be a good place for your discussion of pests from the introduction!

Introduction moved to this location as suggested. Thank you for the recommendation!

General comments:

Is there a specific area that counterfeit products tend to come from? Are there any published isotopic values from these areas?

This depends on the material being sampled and from my personal discussions with chemists in the lab at Oritain and the University of Otago, the origin of counterfeited goods is usually kept private to allow for legal persecution and action to be taken without any complications. I would also find this very interesting to know!

I would love to see a color version of Fig 3!

We can provide a color version for Fig 3. We used a B/W to make it printer and colorblind friendly but I will make a colorblind friendly version and add it to the Supplemental Information.

6. PLOS authors have the option to publish the peer review history of their article ([what does this mean?](https://journals.plos.org/plosone/s/editorial-and-peer-review-process#loc-peer-review-history)). If published, this will include your full peer review and any attached files.


If you choose “no”, your identity will remain anonymous but your review may still be made public.


**Do you want your identity to be public for this peer review?** For information about this choice, including consent withdrawal, please see our [Privacy Policy](https://www.plos.org/privacy-policy).

Reviewer #1: No

Reviewer #2: No

[NOTE: If reviewer comments were submitted as an attachment file, they will be attached to this email and accessible via the submission site. Please log into your account, locate the manuscript record, and check for the action link "View Attachments". If this link does not appear, there are no attachment files.]

While revising your submission, please upload your figure files to the Preflight Analysis and Conversion Engine (PACE) digital diagnostic tool, <https://pacev2.apexcovantage.com/>. PACE helps ensure that figures meet PLOS requirements. To use PACE, you must first register as a user. Registration is free. Then, login and navigate to the UPLOAD tab, where you will find detailed instructions on how to use the tool. If you encounter any issues or have any questions when using PACE, please email PLOS at [figures@plos.org](mailto:figures@plos.org). Please note that Supporting Information files do not need this step.
